# Initial Conditions of Planet Formation: Time Constraints from Small Bodies and the Lifetime of Reservoirs in the Solar Protoplanetary Disk

**DOI:** 10.1007/s11214-025-01216-z

**Published:** 2025-10-17

**Authors:** Maria Schönbächler, Audrey Bouvier, Noriko T. Kita, Thomas S. Kruijer

**Affiliations:** 1 Institute for Geochemistry and Petrology, Department of Earth and Planetary Sciences, ETH Zurich, Clausiusstrasse 25, Zurich, Switzerland; 2 Bayerisches Geoinstitut, Universität Bayreuth, Bayreuth, 95447 Germany; 3 Department of Geoscience, University of Wisconsin-Madison, Madison, WI 53706 USA; 4 Cosmochemical and Isotopic Signatures Group, Lawrence Livermore National Laboratory, Livermore, CA 94550 USA

**Keywords:** Meteorite chronology, Planetesimal formation, Nucleosynthetic data, ^26^Al distribution, Protoplanetary disk, Lifetime of isotopic reservoirs

## Abstract

This review explores the timescales of the initial phase of planet formation, from nebular dust (CAIs and chondrules) to planetesimal accretion and differentiation, using evidence from meteorite research. Aluminium-Mg systematics of CAIs indicate either an extended period of CAI formation (∼0.3 Ma) or an initial ^26^Al heterogeneity, with evidence supporting a homogeneous ^26^Al abundance in the protoplanetary disk. Thermal and aqueous alteration on the parent body can disturb the U-Pb and Al-Mg chronometers in chondrules. Focusing on relatively robust isochron data from plagioclase of pristine (types ≤3.05) chondrites indicates a shift in chondrule formation locations, moving from the inner to the outer disk over time. Ages of basaltic achondrites show that silicate differentiation on small bodies was well underway within the first few million years (Ma) of our solar system. Their age record, however, reveals inconsistencies between different chronometers, partly caused by (i) secondary disturbances due to thermal metamorphism, aqueous alteration, or impacts, (ii) the presence of xenolithic minerals, and (iii) potentially variable initial ^26^Al abundances due to disturbances at the mineral scale. Nucleosynthetic isotope data indicate that parent bodies of iron and stony meteorites formed in two distinct regions within the protoplanetary disk: the inner, non-carbonaceous (NC) and the outer, carbonaceous (CC) region. Based on Hf-W chronometry it has been demonstrated that NC and CC parent bodies of magmatic iron meteorites segregated their cores within ∼1–3 Ma after CAI formation, implying that parent body accretion occurred within <1 Ma in both reservoirs. Combining accretion ages with nucleosynthetic data further reveals that, at first order, NC and CC reservoirs in the solar protoplanetary disk were established within 1 Ma and existed over several Ma with limited exchange between them. In the CR chondrite accretion region of the disk, planetary bodies formed over at least 3 Ma, while in most other regions, formation spanned at least 1 Ma, with minimal changes in nucleosynthetic isotope compositions. Aerodynamical size sorting of dust likely introduced or amplified some of these variations.

## Introduction

The accretion and differentiation of 10-100 km sized planetesimals and larger planetary embryos during the first few million years (Ma) of solar system history represents an essential step towards the growth of larger planetary bodies like the Earth. However, the formation of planetesimals and embryos from small nebular dust constitutes a step in the planet formation process that is notoriously hard to constrain, and the so-called meter-size barrier has been a challenge in models of terrestrial planet formation (Chambers 2004; Nimmo et al. 2018; Raymond and Morbidelli 2022). Vital insights into this problem and, more generally, into planetesimal accretion and differentiation can be obtained from meteorites.

Moreover, the final compositions of planets are influenced at different stages during planet formation: (i) by the composition of dust and gas collected during the infall stage of the disk, (ii) through dust transport and thermal processing (e.g., moving snow line) during the infall stage and within the protoplanetary disk, (iii) through planetesimal formation whose compositions depend on the time and location in the evolving disk, and (iii) finally, by mixing of material during protoplanet growth by collisions of the planetesimals and pebble accretion.

In the past decade, the exploration of early solar system processes has made significant advances in various disciplines. Astronomical observations of protoplanetary disks by the Atacama Large Millimeter Array (ALMA) telescope have provided detailed insights into the structures around new-born stars (e.g., Andrews et al. 2016). Astrophysical models have succeeded in defining new scenarios on how planetesimals and planets are formed (e.g., streaming instabilities and pebble accretion). In cosmochemistry, novel data from meteorites using various isotopic systems have further constrained the timing of dust, planetesimal and planet formation as well as their composition at the different stages.

In this contribution, we will address the initial stages of the protoplanetary disk by reviewing ages of (i) the oldest known material of the solar system preserved in primitive meteorites, i.e. Calcium-Aluminium-rich Inclusions (CAI) and chondrules, (ii) basaltic achondrites, and (iii) magmatic iron meteorites. In contrast to primitive meteorites, basaltic achondrites and iron meteorites originate from differentiated bodies that underwent melting and have preserved crucial evidence about their accretion and differentiation early in solar system history. These bodies experienced partial melting and differentiation to form core, mantle, and crust layers, which can be dated using radioactive decay systems.

### CAIs and chondrules

The elemental abundances and mineralogy of CAIs match those expected for the first ∼5% material that condenses out of a nebular gas of solar composition based on thermodynamic calculations (e.g., Davis and Richter 2014). CAIs are generally assumed to have formed near the Sun, because regions near the young Sun were heated by the energy released from material accreting onto it, leading to the production of solar gas and a transitional region where the gas can cool and condense.

While CAIs have relatively low abundances (<3–10%; Hezel et al. 2008) in chondrites, chondrules are one of the main components (up to 80%; Jones 2012) of primitive chondrites. They are droplets of crystallized silicate melt formed by rapid heating of dust aggregates to high temperatures (>1800 K) followed by hours or days of cooling (e.g., Jones 2012). The formation of CAIs and chondrules occurred in the first few Ma of solar system history, corresponding to the estimated lifetime of the protoplanetary disk (e.g., Evans et al. 2009; Hunt et al. 2022). By definition, the age of CAIs marks the time zero (t_0_) of our solar system, and as such the age of our solar system (e.g., Amelin et al. 2010; Bouvier and Wadhwa 2010; Connelly et al. 2012; Desch et al. 2023b; Piralla et al. 2023). Here, we review recent progress on CAI and chondrule chronology using the U-Pb and Al-Mg decay systems that can resolve small time differences (≤0.1 Ma).

### Basaltic achondrites

These differentiated meteorites are igneous rocks (melts or cumulates) or breccias of igneous rock fragments from the silicate-dominated crust and mantle of large asteroids (i.e., > 20 km in radius) or other planetary bodies (Mars and Moon). The elemental compositions of the basaltic achondrites and their parental melts are fractionated (i.e., enriched in incompatible elements) compared to chondritic compositions (Mittlefehldt 2014). Additionally, impact processes can have modified their compositions by crystal assimilation and brecciation processes (e.g., Rider-Stokes et al. 2023). This complex history needs to be considered when attributing a geological meaning to the ages or for the comparison of ages determined with different chronometers (e.g., K-Ar, Rb-Sr, Sm-Nd, U-Pb) on the same sample. For example, the chronological records of magmatic processes that took place on the meteorite parent bodies can vary across radiometric systems due to differences in elemental diffusion rates. These differences affect the temperature at which diffusion becomes negligible and the system closes, marking the time recorded by each specific decay system. Diffusion rates also contribute to the distinct responses of each decay system to impact-induced changes such as phase transformation, re-heating, and possible re-melting depending on the energy associated with the event and the size of the impacted object (Bogard 1995). As a result, different chronometers may date different events related to the formation and evolution of meteorite parent bodies and ages must be interpreted carefully to accurately link them to the corresponding geological events.

Using a combination of long-lived radiometric (LLR) systems (U-Pb) and short-lived radionuclides (SLR) (Al-Mg, Mn-Cr, Hf-W), the formation of early-formed silicate-enriched reservoirs can be constrained with a precision better than one million years. These decay systems provide complementary information on planetary processes due to the contrasting geochemical behaviour of the parent and daughter isotopes. The Al-Mg system (half-life ∼0.705 Ma) is particularly useful to date silicate differentiation as well as CAIs and chondrules. The Hf-W system (half-life ∼8.9 Ma) is utilised to date metal-silicate differentiation due to W being a siderophile (preserving the W isotopic composition of the core at the time of its formation) and Hf a lithophile element. In addition, the Hf-W system can also date silicate differentiation. The Mn-Cr system (half-life ∼3.7 Ma) provides constraints on nebular processes associated with the volatile nature of Mn ($T_{\mathrm{c}}= 1158\text{ K}$) compared to Cr ($T_{\mathrm{c}} = 1296\text{ K}$) (Lodders 2003) and Mn/Cr fractionation associated with igneous, metamorphic or aqueous alteration processes.

Here we will focus on the chronological record of basaltic achondrites that are used as time anchors to deduce a consistent chronology of early solar system events. Within this framework, we will address the age of CAIs and the magmatic activity on small bodies, which can in turn be used to infer the time of parent body accretion. These age comparisons also prompt the question whether the short-lived nuclide ^26^Al was distributed homogeneously or heterogeneously within our solar system. This has implications for using the Al-Mg decay system for dating, in addition to parent body differentiation (metal-silicate and silicate-silicate), because the radioactive decay of ^26^Al is the major heat source for internal heating in the early solar system (Hevey and Sanders 2006).

### Iron meteorites

Additional vital insights into planetesimal accretion and differentiation come from isotopic studies of iron meteorites. Magmatic iron meteorites consist mainly of metallic iron or Fe-Ni alloys and are interpreted to be fragments of metal cores of protoplanets (Scott 1972; Scott and Wasson 1975). Consequently, magmatic iron meteorites are the only hand specimens of planetary core material in our collection of extraterrestrial samples and provide an opportunity to directly study the differentiation, cooling, and crystallization history of the metallic cores of small differentiated solar system bodies. Trace element signatures of iron meteorites indicate that the metal cores of magmatic iron meteorite parent bodies formed by segregation and crystallization of metallic melts (Scott and Wasson 1975). The classification of iron meteorites, as well as their formation, crystallization, and cooling history, have been discussed in several comprehensive reviews (Chabot and Haack 2006; Goldstein et al. 2009; Benedix et al. 2014; Scott 2020; Bouvier et al. 2025). Here, we focus on accretion and core formation of the iron meteorite parent bodies and their bearing on planetesimal formation and the early evolution of the solar protoplanetary disk. While the accretion time of chondrite parent bodies is constrained by the minimum age of individual chondrules measured in each group, the time of accretion of differentiated meteorite parent bodies has been deduced from geochemical and thermal modelling for bodies internally heated by ^26^Al decay (Hevey and Sanders 2006; Sugiura and Fujiya 2014; Neumann et al. 2018).

### Nucleosynthetic Isotope Variations

**Fig. 1:**
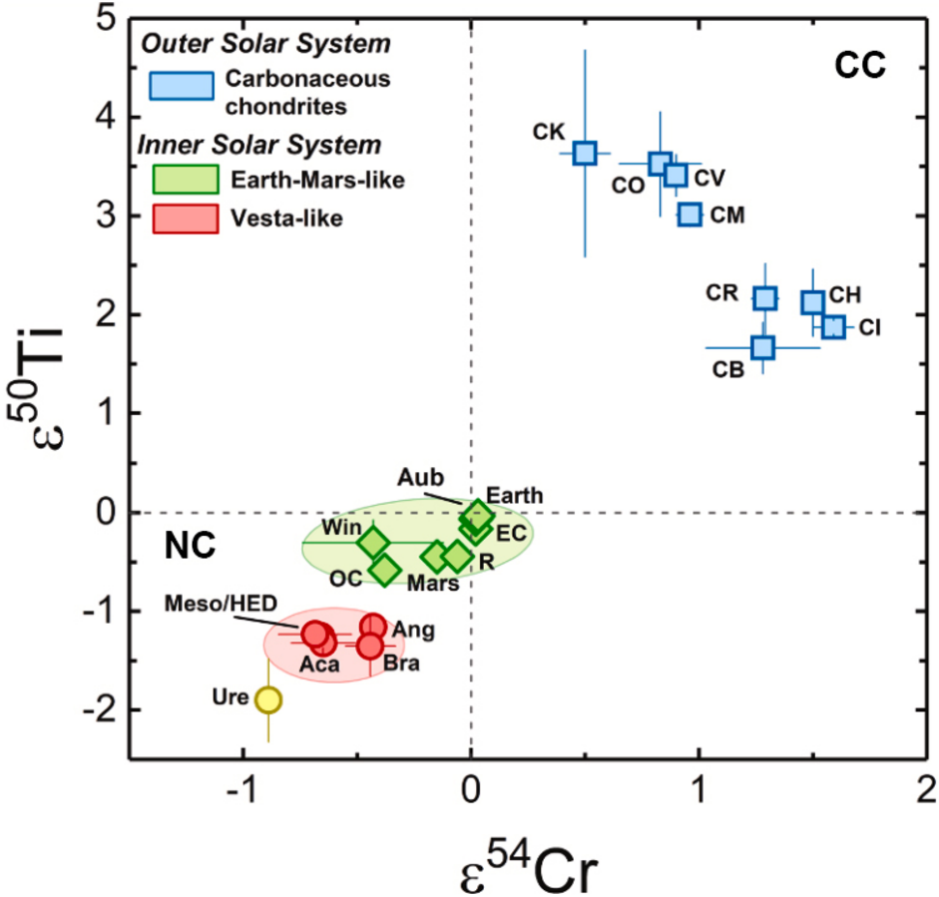
Averages for different meteorite groups for $\varepsilon ^{50}$Ti versus $\varepsilon ^{54}$Cr. In addition to the NC-CC dichotomy (see main text), the NC reservoir displays two distinct clusters (red and green ellipses), which are also distinct from ureilites (yellow). The red and green ellipses comprise the Vesta-like meteorites and the Earth-Mars like meteorites, respectively. Uncertainties are student-t 95% confidence intervals. Epsilon is defined as the deviations of the ^50^Ti/^47^Ti or ^54^Cr/^52^Cr ratio of the sample from the corresponding terrestrial standards expressed in parts per 10,000. Abbreviations: Aub - aubrites, EC - enstatite chondrites, Win - winonaites, OC - ordinary chondrites, R - R-chondrites, Meso - mesosiderites, Aca - acapulcoites, Ang - angrites, Bra - brachinites, Ure - ureilites. From Rüfenacht et al. (2023)

The nucleosynthetic isotope composition of each planetary body is unique, acting as a distinctive identifier for asteroids and planets, with rare exceptions, such as the Earth-Moon system (e.g., Fig. 1). These signatures are powerful tracers to determine genetic relationships between meteorite groups, samples returned by space missions, and planets like Earth and Mars (e.g., Yokoyama et al. 2023; Barnes et al. 2025). They also offer insights into the source regions of meteorites in the solar protoplanetary disk as well as into transport and mixing processes within the disk. Combining nucleosynthetic isotope data with radiometric ages of meteorites and other cosmochemical evidence has proven to be a powerful approach to constrain planet formation. For example, this combination was used to constrain the lifetime of major reservoirs within the solar protoplanetary disk (Warren 2011; Kruijer et al. 2017, 2020), which has important implications for the large scale evolution of the early solar system as well as for the formation timescales and accretion mode of Jupiter (e.g., Morbidelli et al. 2016; Kruijer et al. 2017; Alibert et al. 2018; Brasser and Mojzsis 2020). Additionally, integration of cosmochemical constraints with astrophysical models helped constrain how the evolution of the protoplanetary disk affected the timing and mechanisms of planet formation (e.g., Brasser and Mojzsis 2020; Lichtenberg et al. 2021; Morbidelli et al. 2022; Johansen et al. 2023).

The variations in nucleosynthetic isotope compositions are inherited from stardust (i.e., presolar grains) formed by earlier star generations. These grains, found in primitive chondrites, have extreme and unique isotopic compositions that reflect their stellar formation site. This dust survived in the interstellar medium (ISM) and was mixed into the solar protoplanetary disk together with dust grown in the ISM, the latter with approximately solar system composition (Ek et al. 2020). The incorporation of different amounts of stardust, depending on the formation region in the disk, led to fundamental isotopic differences between all known planetary materials. Depending on the geochemical properties of an element and its carrier phase, parent body processes (e.g., aqueous alteration) can redistribute the nucleosynthetic signal and lead to small scale variations in chondrites (e.g., Cr, Os; Yokoyama et al. 2023, 2011). Additionally, incomplete digestion of presolar grains during bulk sample analysis can produce similar effects (e.g., Schönbächler et al. 2025).

Earlier studies discovered that carbonaceous chondrites exhibit distinctly higher ^54^Cr and ^50^Ti abundances compared to other meteorite groups (Trinquier et al. 2007, 2009; Leya et al. 2008), which is particularly evident when ^54^Cr is plotted versus ^50^Ti (Trinquier et al. 2009). These early studies highlighted two main reservoirs from which early solar system bodies formed: the volatile-rich carbonaceous chondrites that formed outside the snowline and more volatile-depleted material from the inner solar system. These two reservoirs were termed “carbonaceous (CC)” and “non-carbonaceous (NC)” by Warren (2011), who also speculated that carbonaceous chondrites may have formed outside Jupiter’s orbit. These early studies already indicated that these two reservoirs contain both differentiated and undifferentiated bodies based on Ti, Cr and Ni isotope data (Trinquier et al. 2007, 2009; Leya et al. 2008; Steele et al. 2011, 2012). Subsequent work extended the dichotomy to Mo isotopes and further highlighted that the NC-CC dichotomy is not limited to primitive meteorites but also includes differentiated meteorites, including iron meteorites, which are found in both the NC and CC reservoirs (Budde et al. 2016; Kruijer et al. 2017). Among other elements, the dichotomy has been extended to Fe, Ca and Zn (Schiller et al. 2015b; Hopp et al. 2022a,b; Steller et al. 2022; Savage et al. 2022; Martins et al. 2023). How much this dichotomy is reflected in other heavier elements, which show nucleosynthetic isotope variations, can be debated. For iron meteorites, distinct isotope compositions have been reported as evidence for the dichotomy, including siderophile elements such as Ni, Ru, and W (e.g., Kruijer et al. 2017; Nanne et al. 2019; Worsham et al. 2019; Spitzer et al. 2025). Many heavier elements display a continuous trend of nucleosynthetic isotope compositions and have some overlaps of NC and CC materials (e.g., Pd, Nd and W; Worsham et al. 2019; Ek et al. 2020; Frossard et al. 2021; Spitzer et al. 2025). Despite overlaps, the NC and CC data each consistently define their specific regions in the isotope space, highlighting the close genetic relationships within the individual CC and NC reservoirs (Burkhardt et al. 2021; Rüfenacht et al. 2023). In summary, a substantial body of evidence underscores the NC-CC dichotomy as a key feature of solar system formation and evolution. As a first order observation, the solar system material available for laboratory studies can be divided into two types: (i) carbonaceous chondrites and related achondrites, which sample the outer solar system (CC reservoir) and are enriched in supernova-derived isotopes, and (ii) non-carbonaceous chondrites and related achondrite from the inner solar system (NC reservoir), which are generally enriched in $s$-process isotopes.

The preservation of the NC-CC dichotomy indicates an early spatial separation within the solar protoplanetary disk separating the inner and the outer solar system for several Ma (Kruijer et al. 2017; Morbidelli et al. 2022) with minor exchange (e.g., Williams et al. 2020). This in turn requires a dynamical barrier against mixing and homogenization, such as the early formation of Jupiter (Morbidelli et al. 2016; Budde et al. 2016; Kruijer et al. 2017; Alibert et al. 2018), a pressure maximum within the disk (Brasser and Mojzsis 2020) possibly related to the water ice line (Charnoz et al. 2021; Lichtenberg et al. 2021; Izidoro et al. 2022; Morbidelli et al. 2022) or a combination of these processes. The finding that this dichotomy is observed for not only for chondrites, but also iron meteorites and basaltic achondrites provides strong evidence that differentiated meteorite parent bodies formed in the inner (NC) and the outer (CC) protoplanetary disk (e.g., Kruijer et al. 2017).

A closer examination of the nucleosynthetic data reveals isotopic differences within each of the two major reservoirs (NC-CC dichotomy) (e.g., Trinquier et al. 2009; Steele et al. 2012; Akram et al. 2015; Fischer-Gödde et al. 2015; Kruijer et al. 2017; Ek et al. 2020; Frossard et al. 2021; Render et al. 2022). Based on new high-precision Ti and Cr isotope measurements, Rüfenacht et al. (2023) proposed three sub-reservoirs within the inner solar system (NC) reservoir (Fig. 1). They proposed that these sub-reservoirs may mirror disk substructures such as secondary gaps and rings, potentially created by spiral arms emitted from the growing Jupiter and/or Saturn. The distinct isotopic compositions of these reservoirs may result from thermal processing of dust in the disk (Trinquier et al. 2009; Akram et al. 2015; Ek et al. 2020) and/or temporal isotopic variations caused by infalling material from an isotopically heterogeneous molecular cloud (Nanne et al. 2019; Burkhardt et al. 2019) and/or thermal effects during infall (Van Kooten et al. 2016). The variations were likely amplified by dust sorting due pressure bumps and gaps in the protoplanetary disk (Hutchison et al. 2022; Rüfenacht et al. 2023).

In this review, we combine age constraints from CAIs, chondrules and achondrites, with nucleosynthetic isotope data to reassess, whether nucleosynthetic variations reflect (i) the isotopic make-up of their formation location in the solar protoplanetary disk, which remained stable over time or (ii) a signal of evolving isotopic compositions over time in the disk such that each planetary body capture a snapshot of the evolving isotopic composition of the disk at the time of their formation. The goal is to evaluate how long isolated rings can exist in the disk, which is crucial for understanding planet formation. Astronomical observations (ALMA) show rings reflecting dust of >100 μm to mm-size, but we lack information on the ring lifetime, making this an important question to address.

## Chronology of Refractory Inclusions and Chondrules

Formation of CAIs and chondrules in primitive chondrites occurred in the first few Ma corresponding to the lifetime of the protoplanetary disk (e.g., Evans et al. 2009). Here, we focus on recent progress on CAI and chondrule chronology using the U-Pb and Al-Mg systems that can resolve small time differences relevant to the timing and extent of their formation (≤0.1 Ma). The reader may refer to previous review articles, Kita and Ushikubo (2012), Kita et al. (2013) and Nagashima et al. (2018), for comprehensive reviews of earlier studies.

### U-Pb Chronometry

The U-Pb chronometer uses the radioactive decay chains of two U isotopes to two Pb isotopes: ^238^U-^206^Pb, ^235^U-^207^Pb with decay constants $\lambda _{238}$ of 1.55125 × 10^−10^ and $\lambda _{235}$ of 9.8485 × 10^−10^ y^−1^, respectively (Steiger and Jäger 1977). The formation ages ($t$) of CAIs and chondrules provide absolute timescales for their formation events (often referred as “absolute age”) if highly accurate and precise radiogenic (^207^Pb^∗^/^206^Pb^∗^) ratios and U isotope ratios (^235^U/^238^U) of the samples are determined using the Eq. (2.1).

2.1 $$ \frac{^{207} \mathrm{Pb}^{*}}{^{206} \mathrm{Pb}^{*}} = \frac{^{235} \mathrm{U}}{^{238} \mathrm{U}} \times \frac{e^{\lambda _{235} t} -1}{e^{\lambda _{238} t} -1} $$

**Fig. 2:**
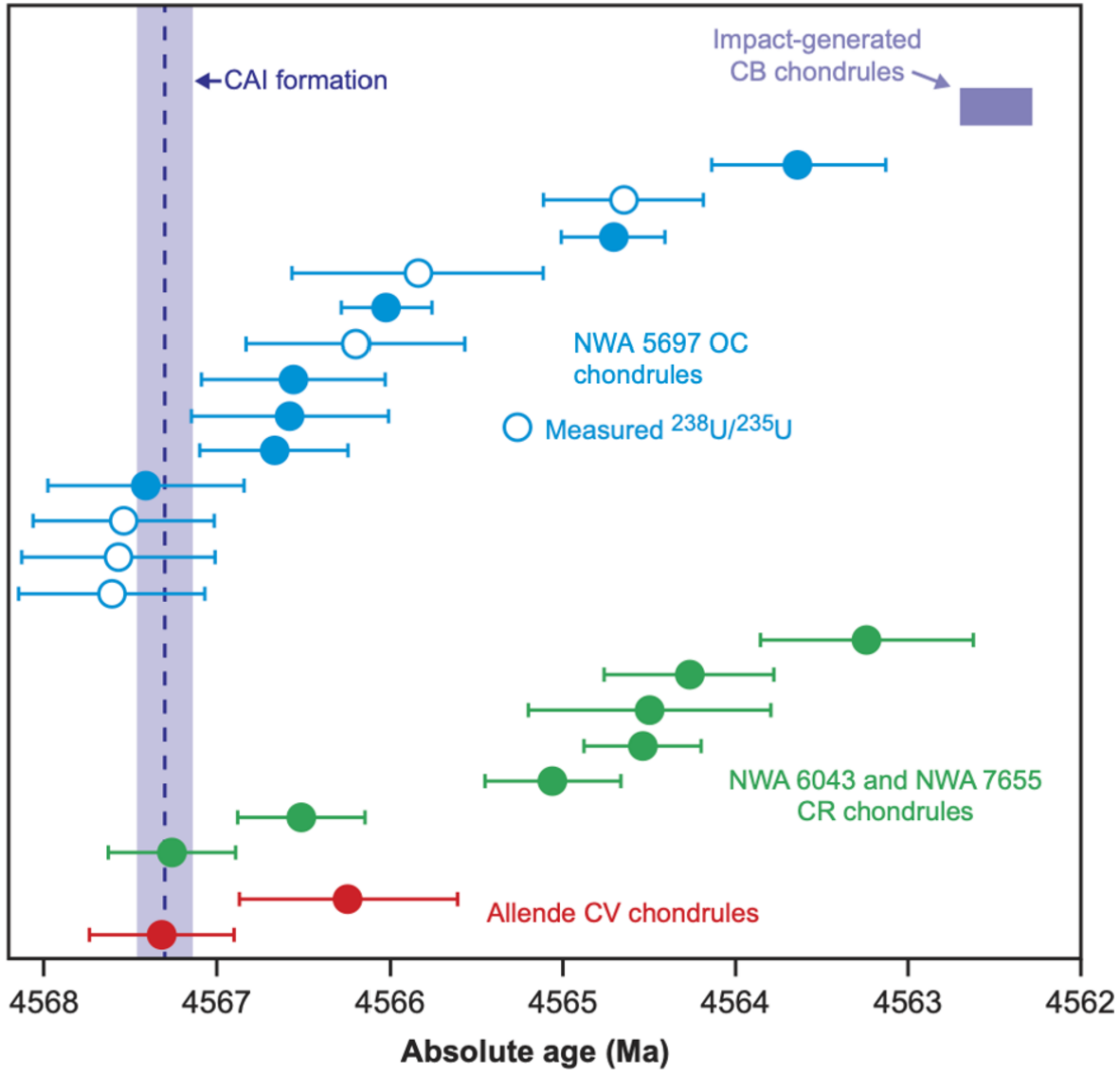
Pb-Pb ages of individual chondrules from ordinary chondrite (NWA 5697, L3.10), CR chondrites and a CV chondrite (Bollard et al. 2017). The shaded area indicates the timing of CAI formation (4567.30 ±0.16 Ma; Connelly et al. 2012). CB chondrule data are shown for comparison (4562.49 ±0.21 Ma; Bollard et al. 2015). The CB chondrules are considered to have formed in a planetesimal impact plume (Krot et al. 2005; Bollard et al. 2015)

The symbol * refers to daughter isotope produced solely by radiogenic ingrowth. Highly accurate estimates of radiogenic ^207^Pb^∗^/^206^Pb^∗^ ratios require samples with extremely low ^204^Pb/^206^Pb ratios of <10^−3^ after the repeated acid leaching procedures, which aim at removing common or unradiogenic (initial or exogenous) Pb (e.g., Amelin 2006). A spread in Pb isotopic compositions is needed to deduce the ^207^Pb^∗^/^206^Pb^∗^ radiogenic ratio from the linear Pb-Pb isochron regression or for two-point model ages using the Pb isotopic composition of troilite from the Canyon Diablo iron meteorite (used as the initial Pb isotopic composition of the solar system because of the absence of U in troilite; Amelin 2006). Variations in ^235^U/^238^U caused by ^247^Cm short-lived radioactive isotope decay in CAIs can bias the Pb-Pb ages up to a few Ma unless accurate U isotope measurements are carried out (Brennecka et al. 2010; Tissot et al. 2016). Published data of CAIs from CV3 chondrites with corresponding U isotope analyses show a small range of U-Pb ages with a mean value of 4567.30 ± 0.16 Ma (n = 5, Amelin et al. 2010; Connelly et al. 2012). A report of an internal U-corrected Pb-Pb isochron on mineral separates of a compact type A CAI from NWA 6991 is, however, notably older at 4567.94 ± 0.31 Ma (Bouvier et al. 2011). Hence, CAI ages show that they are the oldest dated materials in the solar system. In contrast, chondrules from both ordinary chondrites (OC) and CR chondrites show a larger range of Pb-Pb formation ages from 4567.5 Ma to 4563 Ma (Fig. 2, Bollard et al. 2017), suggesting that some chondrules may have formed within ≤0.5 Ma from CAI formation and continued to form for 3-4 Ma, while most chondrules in OC are older than those in CR chondrites.

### Al-Mg Chronology of CAIs

#### Bulk CAI Al-Mg Isochron

**Fig. 3:**
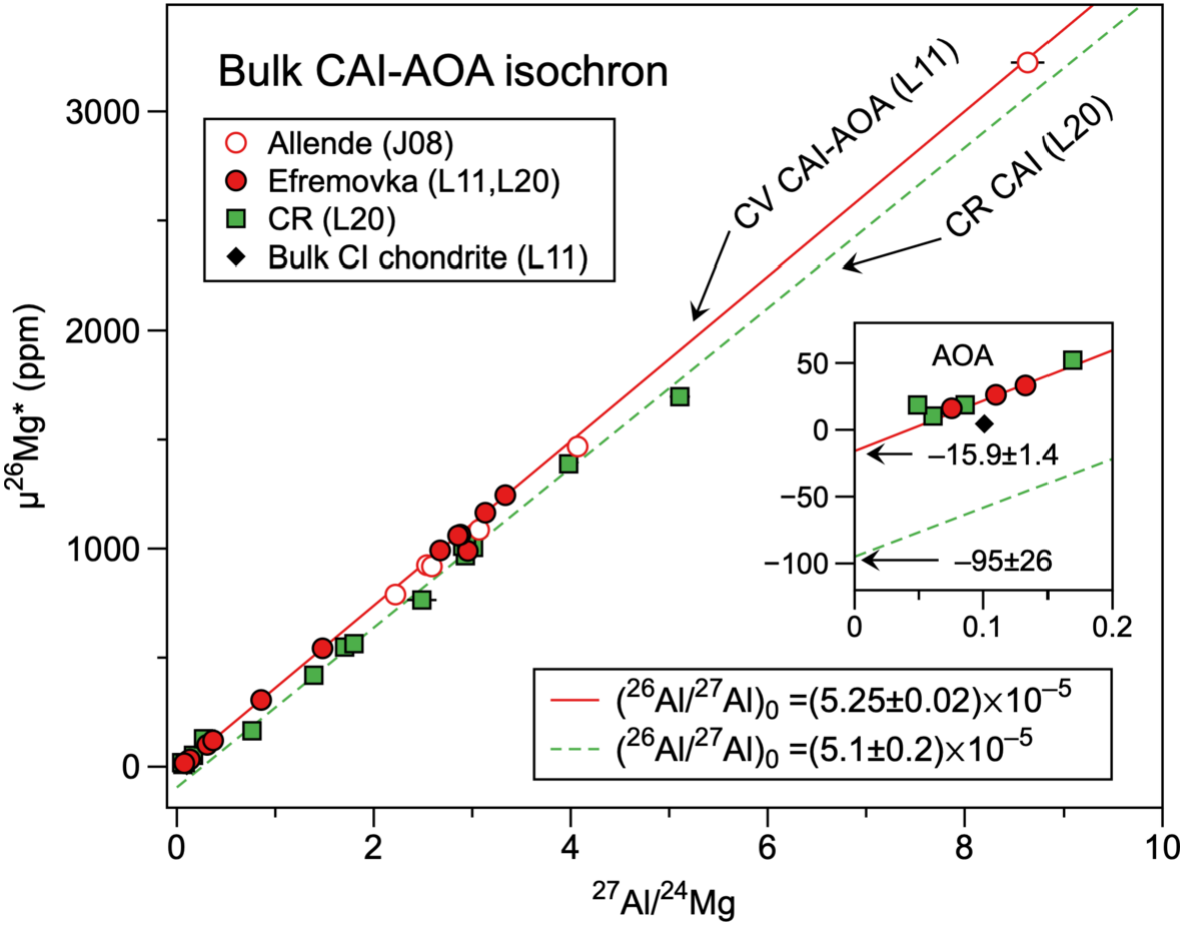
Bulk Al-Mg analyses of CAIs and AOA from CV chondrites (Allende and Efremovka) and CR chondrites (J08 = Jacobsen et al. 2008; L11, L20 = Larsen et al. 2011, 2020). Regression lines are for CV CAI-AOAs (solid line) and CR CAIs (dashed line). The AOAs from CR chondrites (inlet) plot on the same regression line as the CV CAI-AOA isochron

The short-lived nuclide ^26^Al decays to ^26^Mg (half-life $t_{1/2} = 0.705$ Ma; Nishiizumi 2004) and the decay system is used as a chronometer under the assumption of a homogeneous initial ^26^Al abundance in the early solar system (e.g., Kita et al. 2013). Many studies use a half-life of 0.717 Ma which is the weighted mean of literature estimates (Samworth et al. 1972; Norris et al. 1983; Middleton et al. 1983; Thomas et al. 1984). We adopt 0.705 Ma according to Nishiizumi (2004) who estimated the ^26^Al half-life to 0.708 ± 0.017 Ma by using three direct ^26^Al measurements by mass spectrometry (Samworth et al. 1972; Norris et al. 1983; Middleton et al. 1983). This is within uncertainty of the value 0.705 Ma widely used in the cosmogenic nuclide community (Nishiizumi 2004). Earlier studies by Jacobsen et al. (2008) and Larsen et al. (2011) show that multiple bulk Al-Mg analyses of CAIs and amoeboid olivine aggregates (AOA) plot on a single isochron regression line with the slopes corresponding to a (^26^Al/^27^Al)_0_ ratio of ∼5.2 × 10^−5^, which is often referred as the *canonical ratio* of CAIs. The subscript 0 refers to the initial ratio at the time of object formation. More recently, Larsen et al. (2020) reported bulk CAI data from CR chondrites, which also exhibit an indistinguishable (^26^Al/^27^Al)_0_ ratio of 5.1 (± 0.2) × 10^−5^ but with significantly lower initial ($\delta ^{26}$Mg*)_0_ as the intercept of the Al-Mg isochron regression line. Three data sets are summarized in Fig. 3 and suggest that the ^26^Al abundance in the formation location of CAIs seems to be reasonably homogeneous, but Mg isotopes were heterogeneously distributed. Similarly low ($\delta ^{26}$Mg*)_0_ values have been reported from internal isochron studies of individual CAIs, which are likely due to nucleosynthetic anomalies of Mg isotopes (Wasserburg et al. 2012; MacPherson et al. 2017; Larsen et al. 2020). Widespread Mg isotope heterogeneity among CAIs may hinder us from determining the slope of the bulk CAI isochrons with high precision.

Conventionally, we use the (^26^Al/^27^Al)_0_ of 5.252 (± 0.019) × 10^−5^ from CV CAIs determined by Larsen et al. (2011) as that of the initial solar system at time of 0 and estimate a ^26^Al-^26^Mg age relative to CAI formation using the Equation (2.2) below.

2.2 $$ \Delta t_{\mathrm{CAI}} (Ma)= \ln \left [ \frac{\left ( \frac{^{\textit{26}}\mathrm{Al}}{^{\textit{27}}\mathrm{Al}} \right )_{0, \mathrm{CAIs}}}{\left ( \frac{^{\textit{26}}\mathrm{Al}}{^{\textit{27}}\mathrm{Al}} \right )_{0, \mathrm{Samples}}} \right ] \times \frac{0.705}{\ln \left ( 2 \right )}. $$

#### Internal Al-Mg Isochrons of CAIs

**Fig. 4:**
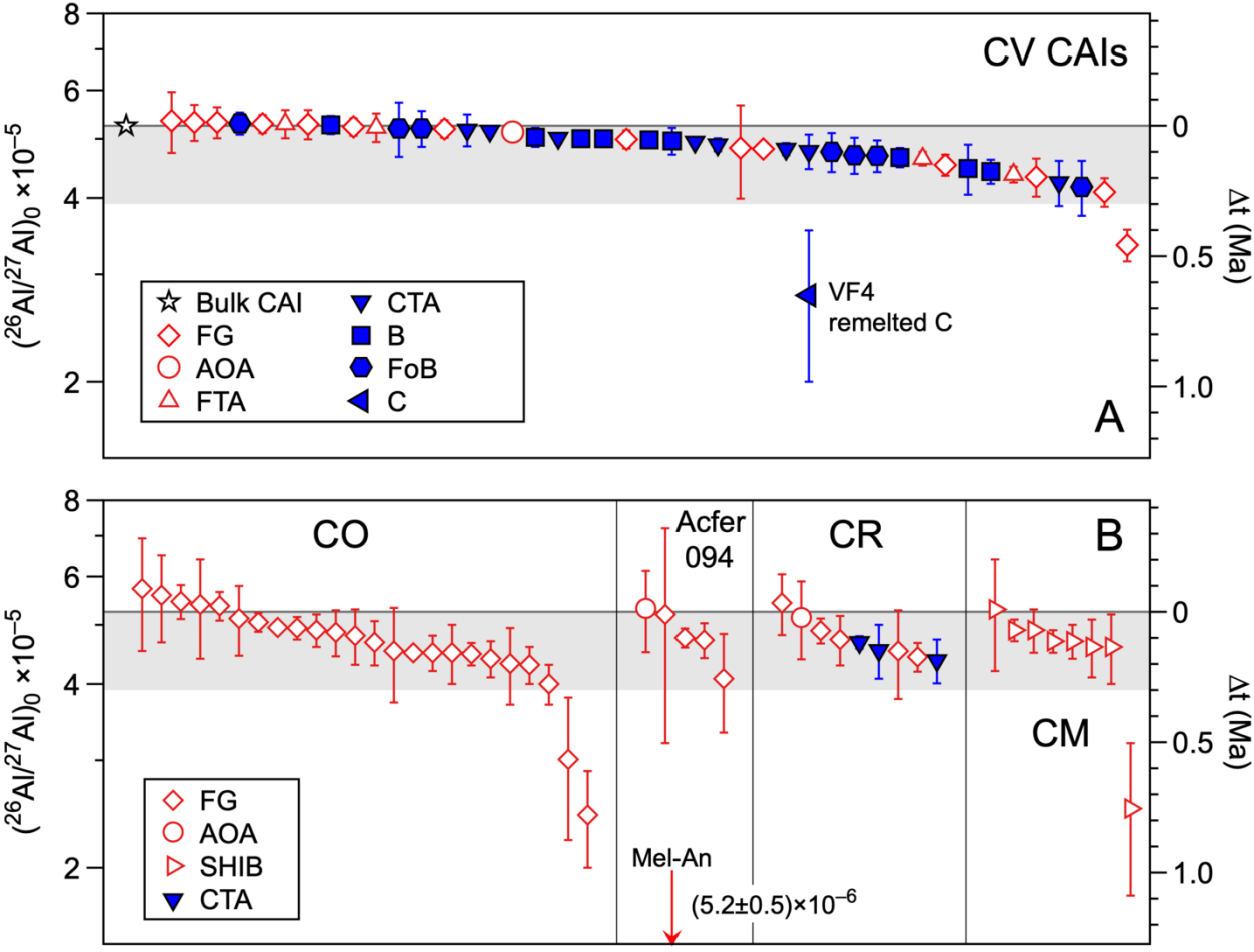
Compilation of initial (^26^Al/^27^Al)_0_ ratios determined from Al-Mg internal isochrons of CAIs and AOAs. The majority of data are indistinguishable from, or within 20%, of the canonical ratios defined by bulk CV CAIs. Two CAIs each from CV chondrites and Acfer 094 show primary and secondary ages. Data source: Jacobsen et al. 2008; Makide et al. 2009; MacPherson et al. 2010, 2012, 2017, 2018, 2020, 2022; Kita et al. 2012; Wasserburg et al. 2012; Kööp et al. 2016b; Kawasaki et al. 2017, 2018, 2019, 2020, 2021; Ushikubo et al. 2017; Liu et al. 2019; Simon et al. 2019; Hertwig et al. 2021; Fukuda et al. 2021; Dunham et al. 2022. Abbreviation of CAI types: AOA: amoeboid olivine aggregate, B: type B, C: type C, CTA: compact type A, FG: fine-grained, FoB: forsterite-bearing type B, FTA: fluffy type A, SHIB: spinel-hibonite. Condensates (AOA, FG, FTA, SHIB) and igneous CAIs (B, FoB, CTA, C) are shown as open and filled symbols, respectively

MacPherson et al. (2012) determined internal Al-Mg isochrons of multiple types of CAIs in CV chondrites and reported uniform (^26^Al/^27^Al)_0_ ratios of ∼5.2 × 10^−5^ for condensate CAIs and a range from 5.2 × 10^−5^ to 4.2 × 10^−5^ for melted CAIs. These results suggested that CAIs experienced high temperature reheating events typically for 0.2 Ma after their initial formation. A summary of (^26^Al/^27^Al)_0_ ratios determined from internal isochrons of CAIs are displayed in Fig. 4 including data from other carbonaceous chondrites. Data from CV chondrites show (^26^Al/^27^Al)_0_ ratios of (4-5) × 10^−5^ regardless of CAI types, such as types A (melilite-rich) and B (melilite-fassaite). Many condensate CAIs, such as Fluffy Type A (FTA) and spinel-rich fine-grained CAIs, exhibit (^26^Al/^27^Al)_0_ consistent with canonical ratios, while others have lower values in the same range as igneous CAIs (e.g., Kawasaki et al. 2017, 2019, 2020). CAIs in Acfer 094, CR, CO and CM chondrites are mostly non-igneous fine-grained CAIs that are likely nebular condensates and reveal a range of (^26^Al/^27^Al)_0_ ratios similar to those of igneous CAIs in CV. These data collectively indicate that condensate CAIs formed contemporaneously with igneous CAIs (e.g., Kawasaki et al. 2021) over ∼0.3 Ma. Several CAIs in Fig. 4 display significantly lower (^26^Al/^27^Al)_0_ ratios suggesting an extended formation and/or reprocessing period of CAIs up to ∼0.7 Ma (MacPherson et al. 2012). In rare cases, CAIs were partially reset ∼2.5 Ma later (Ushikubo et al. 2017).

A small fraction of CAIs exhibits little or no excess $\delta ^{26}$Mg^∗^, suggesting an (^26^Al/^27^Al)_0_ ratio of <5 × 10^−5^ at the time of their formation. These CAIs, often referred to as FUN CAIs, usually have large nucleosynthetic isotope anomalies e.g., in ^48^Ca and ^50^Ti, and mass-dependent fractionation of O, Mg, and Si isotopes (e.g., Kööp et al. 2016a; Park et al. 2017; Williams et al. 2017; Marin-Carbonne et al. 2023). Holst et al. (2013) obtained a well-defined Al-Mg isochron with an (^26^Al/^27^Al)_0_ of 2.9 (± 0.2) × 10^−6^ from a FUN CAI STP-1 in Allende (CV3) that has high (^182^Hf/^180^Hf)_0_ of 0.96 (± 0.11) × 10^−4^, indistinguishable from the initial (^182^Hf/^180^Hf)_0_ ratio of 1.018 (± 0.043) × 10^−4^ of a bulk (non-FUN) CAI isochron, reported by Kruijer et al. (2014a,b). Based on these observations, sub-canonical (^26^Al/^27^Al)_0_ ratios of FUN CAIs are not considered to reflect late formation/reprocessing but instead likely represent early formed CAIs in the protoplanetary disk with a heterogeneous ^26^Al distribution, possibly due to late injection of ^26^Al in the protoplanetary disk or due to selective preservation of presolar isotope signatures (Desch et al. 2023a).

### Al-Mg Chronology of Chondrules

**Fig. 5:**
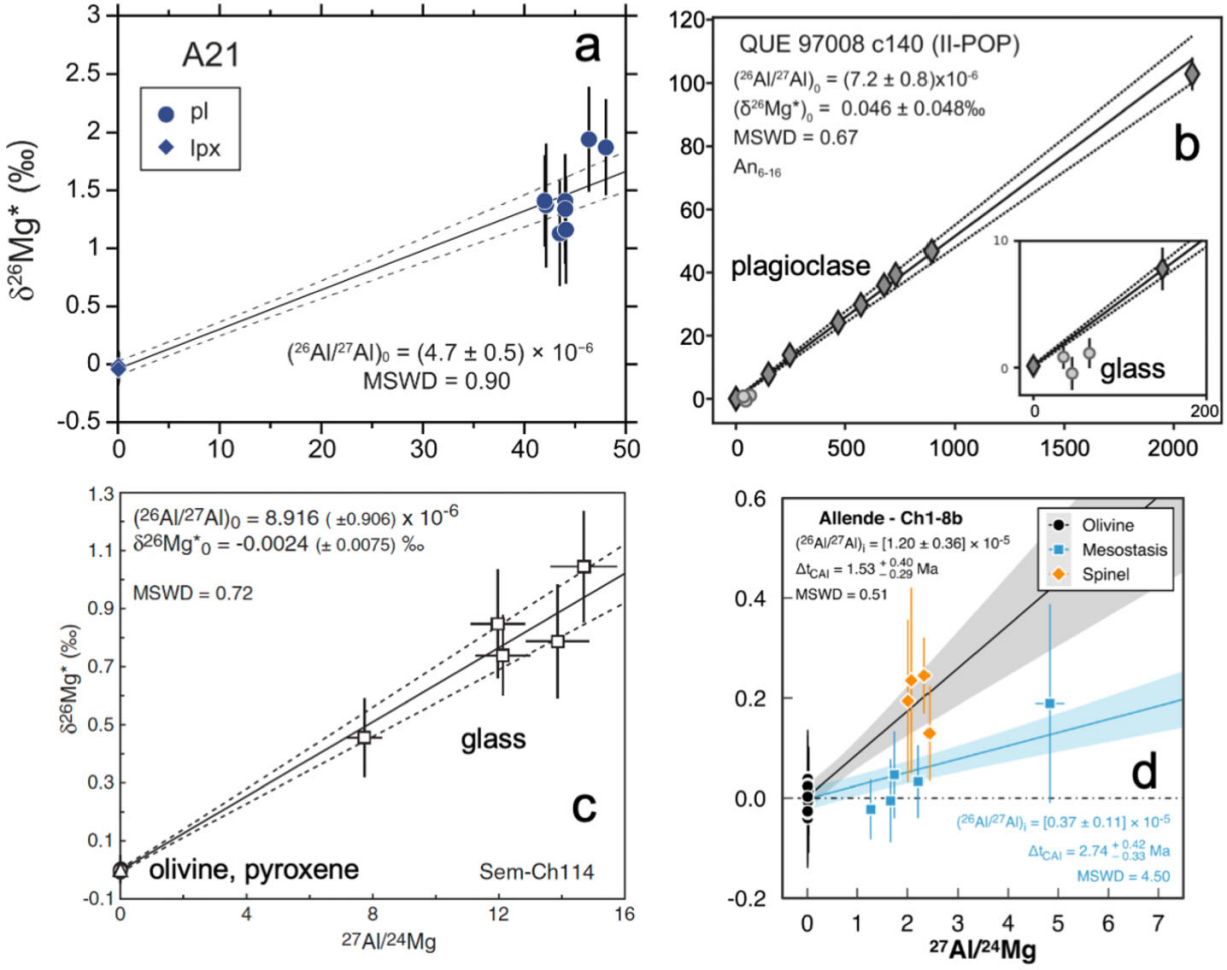
Internal Al-Mg isochrons of chondrules. (a) A 12236 (CM2.9) using anorthite (Fukuda et al. 2022). (b) QUE 97008 (L3.05) using albitic plagioclase and glass (Siron et al. 2022). (c) Semarkona (LL3.00) using glassy mesostasis (Villeneuve et al. 2009). (d) Allende (CV3.6) using spinel and mesostasis (Piralla et al. 2023)

**Fig. 6:**
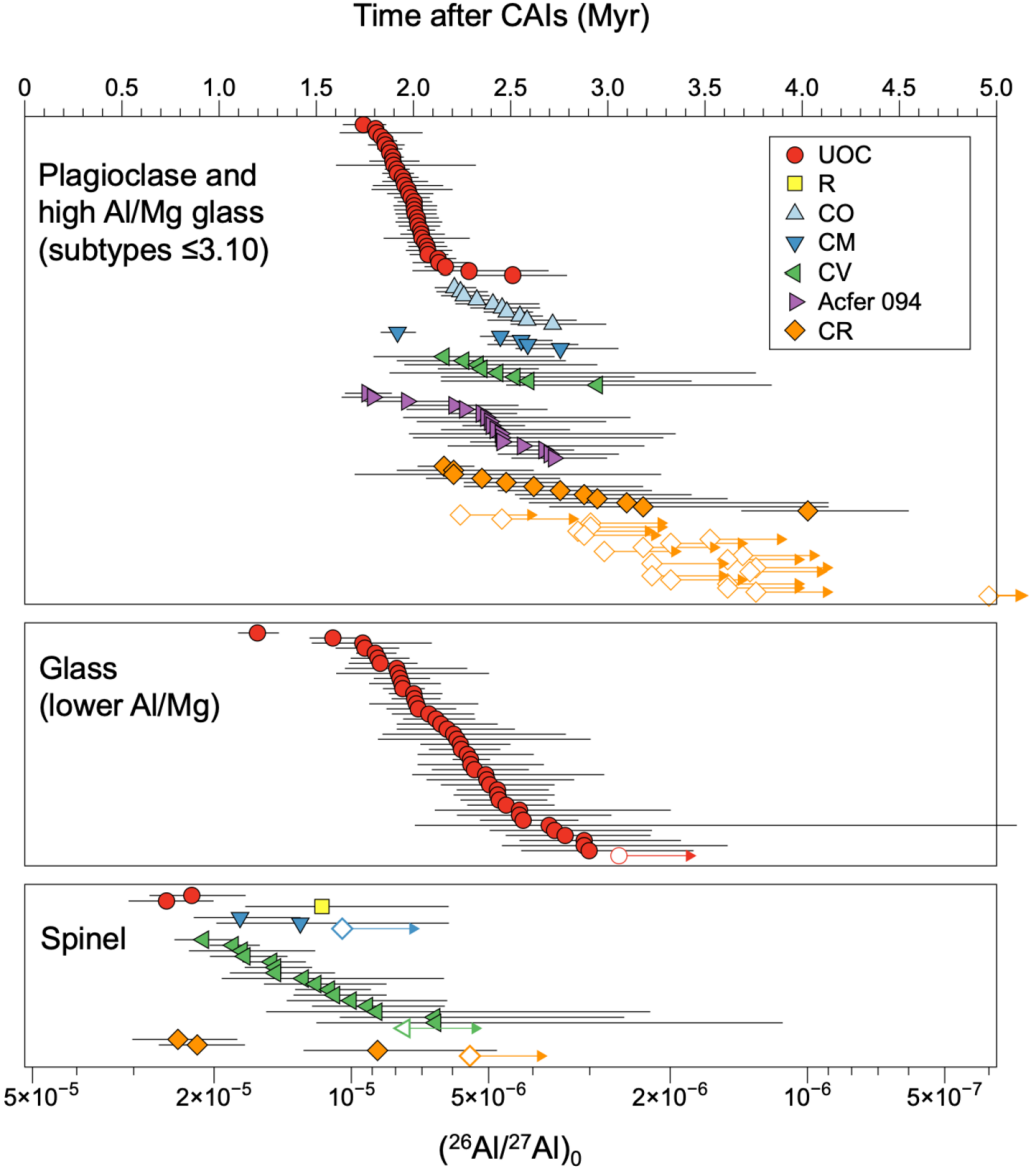
Compilation of Al-Mg data of chondrules obtained from internal isochrons. Open symbols reflect data with upper limit only. Data sources are Ushikubo et al. (2013), Nagashima et al. (2014, 2017), Schrader et al. (2017), Tenner et al. (2019), Bollard et al. (2019), Hertwig et al. (2019), Siron et al. (2021, 2022), Fukuda et al. (2022) for plagioclase and high Al/Mg glass analyses, Villeneuve et al. (2009) and Pape et al. (2019) for low Al/Mg glass analyses, and Piralla et al. (2023) for spinel analyses. UOC - unequilibrated ordinary chondrites

Chondrules predominantly consist of mafic minerals, olivine and pyroxene, and Al-rich phases occur as glassy mesostasis and plagioclase. Spinel is relatively rare but present in chondrules of CV and other carbonaceous chondrites. Most Al-Mg analyses of chondrules were obtained by targeting plagioclase or high Al/Mg glass that show resolvable excess $\delta ^{26}$Mg* (≥1$\permil $) using ≤10 μm small SIMS spot beams (e.g., Siron et al. 2021, 2022). A few studies utilized high precision (≤0.1$\permil $) Mg isotope analyses of Mg-rich mesostasis glass in ordinary chondrite chondrules (Villeneuve et al. 2009; Pape et al. 2019) and spinel crystals in carbonaceous chondrites (Piralla et al. 2023) with smaller excess $\delta ^{26}$Mg* (≤1$\permil $). Examples of Al-Mg isochron diagrams using three different approaches are displayed in Fig. 5. Ideally, such Al-Mg analyses are completed solely on chondrules from the least metamorphosed chondrites (subtypes ≤ 3.10) because Mg diffusion and/or alteration of Al-rich phases even occur at mild metamorphism in the parent body (e.g., Van Orman et al. 2014). A summary of recent results is compiled in Fig. 6.

#### Chondrule Al-Mg Ages Using Plagioclase and High Al/Mg Glass

Plagioclase commonly occurs in chondrules of carbonaceous chondrites, and less commonly in ordinary chondrites. The composition of plagioclase is typically near the pure anorthite endmember for FeO-poor (type I) chondrules in carbonaceous chondrites, while Na-rich plagioclase is found in FeO-rich (type II) chondrules. In OCs, coarse-grained anorthite-bearing chondrules occur with a wide range of FeO compositions in mafic minerals (Siron et al. 2021). They were used in the first Al-Mg study of chondrules and were initially considered to be igneous clasts from a differentiated body (Hutcheon and Hutchison 1989). Self-diffusion of Mg varies significantly with plagioclase composition, with no Mg diffusion at temperatures ≤ 450 °C for anorthite, while the Al-Mg system is significantly affected at 400 °C in albite (LaTourrette and Wasserburg 1998; Van Orman et al. 2014). Kimura et al. (2008) estimated the maximum metamorphic temperatures to be ≤500 °C for metals in chondrites with subtypes ≤3.05. Therefore, the Al-Mg system in anorthite-bearing chondrules from the lowest subtypes (≤3.05) likely remained closed since their formation. In addition, Al-Mg studies of chondrules with albitic plagioclase from subtypes ≤3.05 show well-defined isochrons (Fig. 5b, Siron et al. 2022), suggesting that the Al-Mg system in such chondrules was not disturbed. Siron et al. (2022) also reported a well-defined isochron for 3 chondrules with high Al/Mg glassy mesostasis from type 3.05 chondrites. However, analyses of glass in 2 chondrules with albitic plagioclase plot below the isochron regression lines defined from plagioclase analyses (Fig. 5b). Low degrees of aqueous alterations and thermal metamorphism can modify a part of chondrule mesostasis (e.g., Lewis and Jones 2019), such that careful sample documentation and data evaluation are required for such studies.

**Fig. 7:**
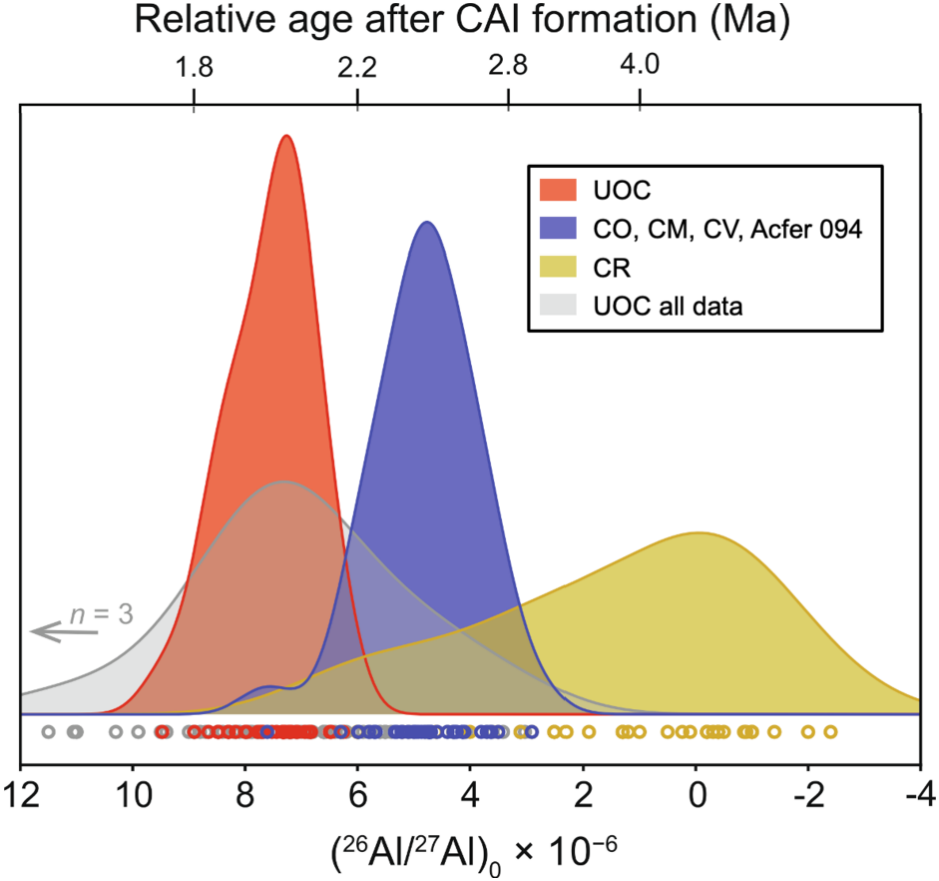
Kernel density estimates of (^26^Al/^27^Al)_0_ ratios for chondrules from UOC, major CC (CO, CM, CV, Acfer 094) and CR chondrites (Fukuda et al. 2022) from SIMS analyses of plagioclase and high Al/Mg glass. Data from all chondrules including low Al/Mg glass analyses are shown as grey area for comparison. Data sources are Ushikubo et al. (2013), Nagashima et al. (2014, 2017), Schrader et al. (2017), Tenner et al. (2019), Hertwig et al. (2019), Siron et al. (2021, 2022), Fukuda et al. (2022) for plagioclase and high Al/Mg glass analyses. For UOC, data include those from Hutcheon and Hutchison (1989), Kita et al. (2000), Rudraswami and Goswami (2007), Rudraswami et al. (2008), Villeneuve et al. (2009), Mishra et al. (2010), Pape et al. (2019), Bollard et al. (2019)

The (^26^Al/^27^Al)_0_ ratios of chondrules determined based on plagioclase and high Al/Mg glass analyses differ systematically among different chondrite groups (Fig. 7). The (^26^Al/^27^Al)_0_ ratio of chondrules using plagioclase and high Al/Mg glass in OC range from (6-9) × 10^−6^ and many of them agree within analytical uncertainties, suggesting that chondrules formed between ∼1.8 and ∼2.1 Ma after CAIs. In particular, Na-rich chondrules studied by Siron et al. (2022) show indistinguishable ages at 2.03 ± 0.10 Ma after CAIs, which is similar to the estimated accretion age of parent bodies of ordinary chondrite using thermal models with ^26^Al as a heat source (∼2.15 ± 0.10 Ma after CAIs; Sugiura and Fujiya 2014; Blackburn et al. 2017). Chondrules in OC forming regions accreted immediately (≤0.1 Ma) to the parent bodi(es), consistent with the formation of OC chondrules in highly dense disk regions (Alexander et al. 2008). The (^26^Al/^27^Al)_0_ ratios of chondrules from CO, CM, CV, and Acfer 094 chondrites are mostly (3-6) × 10^−6^, corresponding to formation ages of 2.2–2.8 Ma after CAIs. A few chondrules from Acfer 094 and CM chondrites exhibit higher (^26^Al/^27^Al)_0_ ratios and older formation ages, similar to those in OC and their O isotope signatures, and mineral compositions are also similar to OC (Hertwig et al. 2019; Fukuda et al. 2022). In CR chondrites, a majority of the chondrules lack resolvable excess $\delta ^{26}$Mg* with an upper limit of (^26^Al/^27^Al)$_{0}\ \leq $3 × 10^−6^ (Nagashima et al. 2014; Schrader et al. 2017; Tenner et al. 2019), which is consistent with relatively late formation of chondrules in CR chondrites as indicated by their Hf-W systematics (Budde et al. 2018). A small subset of CR chondrules with resolvable excess $\delta ^{26}$Mg* shows similarities to chondrules in CO, CM, CV, and Acfer 094 chondrites in terms of the range of (^26^Al/^27^Al)_0_, O isotope ratios, and mineral chemistry, suggesting that they might have formed in a similar environment or share a common origin (Tenner et al. 2019).

Fukuda et al. (2022) argue that chondrule formation started from the inner disk and that the formation locations moved to the outer disk with time; at ∼ 2 Ma (after CAIs) in the OC chondrite forming region, at 2.2–2.8 Ma in the major carbonaceous chondrite forming region, and at ≥2.8 Ma in the CR chondrite forming region. It is interesting to find older chondrules with NC-like oxygen isotope signatures in CM and Acfer 094 (Fukuda et al. 2022; Hertwig et al. 2019) as well as “CO-like” (Marrocchi et al. 2022) older chondrules in CR chondrites (Tenner et al. 2019). Their presence may indicate the transport of chondrules from the inner to the outer disk, where older chondrules became a part of chondrule precursors (e.g., Hertwig et al. 2019; Schrader et al. 2020; Williams et al. 2020). However, the early formation of proto-Jupiter (≤1 Ma after CAIs; Kruijer et al. 2017) might have hampered the outward transport of chondrule-sized objects. Alternatively, the isotope signatures and environments of later forming chondrules had changed locally within individual disk regions (e.g., Tenner et al. 2019; Marrocchi et al. 2022).

#### Chondrule Al-Mg Ages Using Low Al/Mg Glass

Villeneuve et al. (2009) and Pape et al. (2019) conducted high precision Mg isotope analyses (precisions ≤0.1$\permil $) of chondrules in OC (subtypes 3.01-3.10) by targeting glassy mesostasis with low Al/Mg ratios using large SIMS spots (∼30 μm). Their reported range of (^26^Al/^27^Al)_0_ overlaps with those determined for a similar suite of meteorites by Siron et al. (2021, 2022), who analysed plagioclase and high Al/Mg glass. However, analyses of low Al/Mg mesostasis define a wider range of (^26^Al/^27^Al)_0_ ratios from ∼1.6 × 10^−5^ to ∼3 × 10^−6^, corresponding to 1.2 Ma to 3 Ma after CAIs. In addition, two chondrules do not show correlated $\delta ^{26}$Mg* excesses and a slope of ∼0. The majority of analyses were obtained on the fine-grained mixture of glassy mesostasis and Mg-rich micro-crystallites, in which radiogenic ^26^Mg might have been redistributed. However, a deviation from the isochron regression lines cannot be identified by statistical evaluation due to the generally small $\delta ^{26}$Mg* excess (typically ≤0.3$\permil $). Alternatively, Pape et al. (2021) argue that the younger ages of ∼3 Ma after CAIs represent the timing of remelting based on detailed petrological examination of multi-layered chondrules.

#### Chondrule Al-Mg Ages Using Spinel

Spinel (MgAl_2_O_4_) is rare in chondrules. The majority of the Al-Mg data obtained on spinel are from chondrules of CV chondrites because these meteorites contain spinel more frequently than other chondrite groups. However, spinel in CV chondrites could be metamorphosed (loss of radiogenic ^26^Mg or Fe-Mg exchange by diffusion) and/or be a relict phase from CAI and AOA precursors that already accumulated radiogenic ^26^Mg prior to chondrule formation. Therefore, Piralla et al. (2023) restricted their Al-Mg analyses of spinel to FeO-poor (type I) chondrules without metamorphic zoning in Fe-Mg, and with O isotope ratios in agreement with olivine in the same chondrule. The resulting Al-Mg ages (Fig. 6) are systematically older than those using plagioclase and glass by ∼1 Ma. Piralla et al. (2023) argue that the self-diffusion in spinel is slower than in plagioclase and glass, such that the Al-Mg age determination using spinel is the more robust approach. They concluded that chondrules started to form ∼0.8 Ma after CAIs and continued to form for 2-3 Ma during the entire lifetime of the protoplanetary disk. Such a long duration of chondrule formation is in principle consistent with the range in U-Pb ages reported for individual chondrules (Bollard et al. 2017). However, whereas the Al-Mg ages reveal a clear time gap between CAIs and chondrules, this is not evident from the U-Pb ages of individual chondrules.

As discussed earlier, the self-diffusion rate of Mg in plagioclase varies with composition, and in anorthite, which commonly occurs in type I chondrules, is actually similar to that of spinel (Sheng et al. 1992; LaTourrette and Wasserburg 1998; Van Orman et al. 2014). Major element compositions of anorthite-rich plagioclase in types ≤3.05 chondrites show silica-excesses and high abundances of MgO (0.5-1%), likely preserving Mg since their formation at high temperatures. Therefore, the systematic difference in Al-Mg ages between spinel and anorthite-rich plagioclase in type I chondrules is not expected for the least metamorphosed ≤ 3.05 chondrites. Spinel Al-Mg ages obtained by Piralla et al. (2023) are mainly from CV chondrites (subtypes ≥3.1), which display significant variations in MgO and FeO (Schnuriger et al. 2022), likely due to Fe-Mg diffusional exchange. MacPherson et al. (2012) reported compelling evidence for the re-distribution of ^26^Mg excesses between anorthite and spinel in Vigarano (CV3) type B CAI “F1” due to parent body thermal metamorphism. In this CAI, the anorthite likely lost radiogenic ^26^Mg due to thermal metamorphism ($\delta ^{26}$Mg$^{*}\ \leq $10$\permil $), which was redistributed into spinel enclosed in anorthite with the $\delta ^{26}$Mg^∗^ values 1-2$\permil $ higher than the isochron regression line defined by melilite, fassaite, and spinel enclosed in melilite and fassaite. Self-diffusion of Mg in spinel is comparable to that in anorthite and the Fe-Mg inter-diffusion rate is even faster (Van Orman and Crispin 2010; Van Orman et al. 2014). In chondrules, spinel is often in contact with mesostasis (Schnuriger et al. 2022) that could have higher ^27^Al/^24^Mg ratios than spinel (> 2.5). During parent body metamorphism, radiogenic ^26^Mg is likely re-distributed in a similar manner as in type B CAI F1 resulting in $\delta ^{26}$Mg^∗^ enhancements. Among the chondrules studied by Piralla et al. (2023), only two were from subtypes 3.00-3.01 chondrites and both show very high (^26^Al/^27^Al)_0_ of ∼2 × 10^−5^; Ch1 in Acfer 094 (ungroup C3.00) and Ch10 in Semarkona (LL3.01). Analyses of glass in Ch10 (Semarkona) do not show resolvable ^26^Mg excesses, possibly the excesses were re-distributed by very low degrees of metamorphism, although Mg isotope exchange between spinel and glass is unlikely. Oxygen isotope ratios of Ch1 of Acfer 094 exhibit large variations in $\delta ^{18}$O and $\delta ^{17}$O along a slope 1 line with spinel being more ^16^O-rich than olivine. This suggests that spinel is a relict phase from CAIs. For Ch10, no O isotope data are available, such that it is unclear if spinel in Ch10 also represents a relict from CAIs. To clarify this issue, further studies are required for spinel in chondrules combining Al-Mg chronology with detailed petrography, mineral chemistry, O isotope ratios, and Mg isotope analysis of mesostasis.

### Disagreements of U-Pb and Al-Mg Ages Among CAIs and Chondrules

The initial (^26^Al/^27^Al)_0_ values of most CAIs vary as much as 20-30% from the canonical value, suggesting either the extended period of CAI formation (∼0.3 Ma) or heterogeneity of initial ^26^Al abundance among CAIs. Such short formation intervals among CAIs are consistent with a narrow range of U-Pb ages of CAIs (4567.30 ± 0.16 Ma; n = 5, Amelin et al. 2010; Connelly et al. 2012). Initial (^26^Al/^27^Al)_0_ ratios of chondrules determined from plagioclase isochrons in pristine chondrites (types ≤3.05) suggest systematic changes in the chondrule formation location with time, possibly related to growth of protoplanets. However, U-Pb ages of chondrules show a significantly larger range compared to those of Al-Mg ages. Both the U-Pb and Al-Mg systematics of CAIs and chondrules might be easily disturbed by a low degree thermal and aqueous alteration. Earlier formation of chondrules (<1.7 Ma after CAIs) cannot be fully ruled out, although the chronometers were likely disturbed due to heat generated in the parent bodies.

## The Importance of a Detailed Chronology of Basaltic Achondrites

Basaltic achondrites (excluding martian and lunar meteorites) are mafic igneous rocks or breccias composed of igneous rock fragments from the crust and mantle of large asteroids. The parent bodies of basaltic achondrites experienced partial melting and differentiation into a core, mantle, and crust. The basaltic achondrites and their parental melts are enriched in incompatible elements compared to chondritic compositions (Mittlefehldt 2014).

The chronological records of crust-mantle differentiation and secondary processes such as impact events can be obtained from LLR systems (e.g., K-Ar, Rb-Sr, Sm-Nd, U-Pb). Short-lived radionuclide systems (e.g., Al-Mg, Mn-Cr, Hf-W), with their shorter half-lives, generally provide better age precision. The chronological records of meteorites can be selectively modified across different radiometric systems due to the varying diffusion behaviours of elements during thermal events. Despite limited constraints on diffusion parameters associated with chemical species of mineral hosts of interest (e.g., Cr diffusion kinetics in spinel, Posner et al. 2016; Mg diffusion in anorthite, LaTourrette and Wasserburg 1998; Van Orman et al. 2014), individual age dates from LLR or SLR systems provide important constraints on the thermal history of rock samples from crystallisation to impact events. The individual dates can serve as input for numerical models to constrain the accretion time and size of achondrite parent bodies (e.g., Golabek et al. 2014; Neumann et al. 2018). An assessment of the significance of the individual dates needs, however, to be carried out because some chronometers may be affected by secondary processes, hence, indicating a secondary event or possibly a partial reset; the latter implies that the age does not hold geological significance. If the dates from several radiometric systems correspond to rapid cooling of the magmatic system and have not been altered by secondary processes, they can be compared across different meteorites formed in distinct regions of the protoplanetary disk. This comparison helps constrain the initial abundances and distribution of SLR such ^26^Al (e.g., Schiller et al. 2015a; Desch et al. 2023b).

### Timescales of Silicate Differentiation

The timescales of the formation of silicate-enriched reservoirs can be constrained with a precision below 1 Ma by using the two long radioactive decay chains of ^238^U($\rightarrow ^{206}$Pb) and ^235^U($\rightarrow ^{207}$Pb) isotopes to deduce ages based on the radiogenic ^207^Pb*-^206^Pb* compositions. The radiogenic ^207^Pb*/^206^Pb* ratio provides the highest precision (at best ±100,000 years, Amelin and Yin 2025) for LLR systems, but it requires the measurement and use of the corresponding ^238^U/^235^U ratio of planetary materials. The ^238^U/^235^U ratio can vary due to radioactive decay of ^247^Cm (half-life ∼15.6 Ma) or due to mass-dependent isotopic fractionation during chemical reactions and secondary processes (which can be significant if associated with a change in U redox state) (Brennecka et al. 2010; Goldmann et al. 2015; Tissot et al. 2016; Huyskens et al. 2025). The ages can be combined with high-resolution chronology using SLR. Two SLR systems are particularly useful to date silicate differentiation. These are (i) the ^26^Al-^26^Mg system that provides constraints on condensation, melting and crust-mantle formation due to contrasting condensation temperatures ($T_{50}= 1653\text{ K}$ for Al and $T_{50}= 1336\text{ K}$ for Mg) and compatibility in felsic or mafic minerals; and (ii) the ^53^Mn-^53^Cr system that provides insights into nebular processes owing to the volatile nature of Mn ($T_{50} = 1158\text{ K}$) compared to Cr ($T_{50} = 1296\text{ K}$) (Lodders 2003) as well as Mn/Cr fractionation linked to igneous, metamorphic, or aqueous alteration processes. While the short-lived ^182^Hf-^182^W system has also provided key constraints on silicate differentiation history of planetesimals, the focus of this section is explicitly on constraints provided by the ^53^Mn-^53^Cr and ^26^Al-^26^Mg systems.

Primitive (e.g., brachinites, acapulcoites) and basaltic achondrites (e.g., basaltic eucrites, quenched angrites, NWA 6704 grouplet), have early crystallisation ages within the first 10 Ma of solar system history (e.g., see Bouvier et al. 2025 for a review). Some achondrites even pre-date chondrule formation in the NC region such as Erg Chech 002 (EC 002) ungrouped achondrite, with reported Al-Mg ages ranging from 1.8 to 2.1 Ma after CAIs (Barrat et al. 2021; Reger et al. 2023; Connelly et al. 2023) (see further discussion on EC 002 chronological records in Sect. 3.3). Later crystallisation ages, up to 50 Ma after CAIs, are found for the cumulate eucrites and indicate an extended period of plutonic magmatism on Vesta (e.g., Bouvier et al. 2015; Roszjar et al. 2016). Such a prolonged period of magmatism was also recently reported from younger Pb-Pb ages of Ca-phosphates at 4514 ± 30 Ma that were found in the dunitic cumulative angrite NWA 8535 (Rider-Stokes et al. 2024). Later major impact events were also identified, e.g., responsible for the mesosiderite formation at 4525.39 ± 0.85 Ma (Haba et al. 2019).

### Chronological Anchors for Short-Lived Radionuclides

The U-isotope-corrected Pb-Pb ages of rapidly cooled and pristine (unshocked) basaltic achondrites are the only radiometric ages in absolute time scale that can be obtained with high enough precision to be compared to the relative SLR ages (e.g., Brennecka and Wadhwa 2012). They are, however, dependent on knowing precisely the U isotopic composition of the analysed mineral (e.g., Brennecka et al. 2010; Reger et al. 2023). There should also be an absence of secondary disturbance by planetary or weathering processes that are commonly observed in meteorite finds (Tissot et al. 2017; Bouvier et al. 2025). If undisturbed, the U-corrected Pb-Pb ages of individual meteorites may then be compared against relative SLR age differences between these relative chronometers and to model the initial abundance of the respective radioactive isotopes at a given time; traditionally taken as t_0_, the time of condensation of refractory inclusions (i.e., CAIs) and formation of the solar system (e.g., Desch et al. 2023b).

A particular petrological suite of achondrites has been used as a potential reference point for relative ages determined by various SLR systems, including the Al-Mg, Mn-Cr, and Hf-W decay systems. The angrites, in particular the volcanic types (e.g., D’Orbigny and Sahara 99555), formed around 4 to 5 Ma after CAIs (Brennecka and Wadhwa 2012; Tissot et al. 2017). They cooled rapidly after crystallization, facilitating synchronized records across different chronometers, because the time for diffusion was very limited. The volcanic angrites generally lack reports of significant major shock effects, which could have altered their primary chemical and isotopic compositions. Recently, however, moderate to high shock effects were reported for NWA 1670 (containing olivine xenocrysts with fractures, mosaicism and undulatory extinction) and NWA 7203 (melt shock veins associated with amorphous anorthite and younger Pb-Pb ages of silicophosphates at 4543 ± 19 Ma) (Hayashi et al. 2022). Distinct isotopic compositions in $\Delta ^{17}$O in olivine xenocrysts of several angrites support isotopic mixing (Rider-Stokes et al. 2023). The higher initial ^26^Al abundance in spinel compared to plagioclase in D’Orbigny indicates a disturbance of the Al-Mg chronological records in this quenched angrite, and questions its use as an anchor for SLR systems (Deligny et al. 2024).

Plutonic angrites formed approximately 7 Ma after their volcanic counterparts, after the extinction of ^26^Al (hence no Al-Mg ages are reported), and experienced slower cooling, as evidenced by their coarse-grained textures (Brennecka and Wadhwa 2012; Datta et al. 2024). The diabasic angrite NWA 12320 has pyroxene with a Pb-Pb age of ∼4564 Ma that is similar to quenched angrites, and 1.3 ± 0.6 Ma younger U-Pb phosphate ages suggesting rather slow cooling or later reheating (Datta et al. 2024). The U-corrected Pb-Pb ages of both quenched and plutonic angrites align with their Hf-W and Mn-Cr systematics and this indicates a homogeneous distribution of ^182^Hf and ^53^Mn in the formation regions of CAIs and the angrite parent body (Kleine et al. 2012; Kruijer et al. 2014a,b; Zhu et al. 2019). Falling between the quenched and plutonic angrites, a group of brecciated angrites, including NWA 2999 and NWA 6291, shows a mix of coarse- and fine-grained textures and metal-rich lithologies. The ^147^Sm-^143^Nd age of NWA 2999 angrite is 3925 ± 125 Ma (Sanborn et al. 2015), which is significantly younger than its U-isotope corrected Pb–Pb age of ∼4560 Ma (Brennecka and Wadhwa 2012) and the ages provided by the short-lived Hf–W and Mn–Cr chronometers (Shukolyukov and Lugmair 2008; Kleine et al. 2012).

The initial ^182^Hf/^180^Hf ratio of the solar system was determined at (1.018 ± 0.043) × 10^−4^ based on a Hf-W isochron of bulk CAIs (Kruijer et al. 2014a,b). The solar system’s ^53^Mn/^55^Mn_0_ ratio cannot be directly measured in CAIs, and was estimated at (7 ± 1) × 10^−6^ using U-corrected Pb-Pb ages and SLR systematics of quenched and plutonic angrites, with a relatively large uncertainty due to the variability in CAI U-corrected Pb-Pb ages (Tissot et al. 2017; Sanborn et al. 2019). Early igneous activity on planetesimals suggests that ^26^Al was the major radioactive heat source for the first few Ma of solar system history. The initial ^26^Al abundance is a critical parameter to constrain numerical models of planetary accretion and differentiation. The initial and canonical abundance of ^26^Al was unambiguously determined from tens of individual and mineral isochrons of “normal” CAIs to be ∼5.2 × 10^−5^ (Jacobsen et al. 2008; MacPherson et al. 2010; Larsen et al. 2011, 2020) (see Sect. 2.2 on CAIs). Discrepancies in estimating the initial ^26^Al abundance have been found when comparing the ^26^Al-^26^Mg and U-corrected Pb-Pb ages for individual CAIs against single (CR or LL) chondrules or achondrites (NC angrites, Asuka 881314, Erg Chech 002, and CC NWA 2976 and NWA 6704 grouplets) (Schiller et al. 2015a; Bollard et al. 2019; Sanborn et al. 2019; Wimpenny et al. 2019; Connelly et al. 2023; Krestianinov et al. 2023; Reger et al. 2023). From chronological studies of the SAH 99555 and D’Orbigny angrites as well as Erg Chech 002, it was suggested that ^26^Al could be heterogeneously distributed with variable degrees of depletion, up to a factor of 3 to 4 compared to the canonical value, and within the NC formation region (Connelly et al. 2023; Krestianinov et al. 2023). It was proposed that this heterogeneous distribution within the protoplanetary disk was subsequently modified over time by inward-moving ^26^Al-rich dust of solar CI composition from the outer to the inner disk (Schiller et al. 2015a,b; Bollard et al. 2019). If ^26^Al was present in abundance lower than the canonical value (∼5.2 × 10^−5^), such as the abundances that have been reported in NC achondrite forming regions (i.e., (1.5-2.5) × 10^−5^) and ^26^Al decay was the primary energy source for planetesimal heating, it would restrict the period of accretion of differentiated planetesimal to within the first 100,000 yr after CAIs to reach melting temperature and a magma ocean stage (Schiller et al. 2015a; Bollard et al. 2019). If so, an ^26^Al difference between unrelated objects would not have any chronological meaning (Bollard et al. 2019).

Taking a closer look at angrite isotopic records, two independent ^26^Al-^26^Mg studies of the D’Orbigny angrite suggested a possible age adjustment of 0.3 ± 0.1 Ma towards younger ages, which corresponds to a ∼20% difference in the calculated initial abundance (Spivak-Birndorf et al. 2009; Schiller et al. 2015a,b). Uranium isotopic compositions vary between quenched and plutonic angrite groups and evidence of non-secular equilibrium values in ^234^U associated with U remobilization during terrestrial alteration is also reported in angrites (most angrites being desert finds) (Tissot et al. 2017). Differences in U isotopic compositions were identified between acid-washed minerals (e.g., pyroxenes) and phosphates in plutonic angrites, but not in all angrites (Datta et al. 2024; Huyskens et al. 2025). The U-corrected Pb-Pb age of the quenched angrite D’Orbigny does not seem to be affected by U isotopic effects considering mineral or whole-rock fractions (Huyskens et al. 2025). Angra dos Reis and NWA 4590 have, however, lower ^238^U/^235^U ratios of ∼137.78 for leached or unleached whole-rock samples compared to ∼137.80 and 137.83 for pyroxene residues and phosphates, respectively, that should be used to correct the Pb-Pb ages of each corresponding phase (Huyskens et al. 2025; Tissot et al. 2017). In contrast, the diabasic angrite NWA12320 does not show such an effect between whole-rock and acid washes containing soluble phosphates (Datta et al. 2024). This inter-mineral variation for U isotopes was also measured between leached pyroxene fractions (^238^U/^235^U = 137.77 ± 0.03) used for the Pb-Pb isochron regression and whole-rock compositions (^238^U/^235^U = 137.82 ± 0.01) of Erg Chech 002, leading to a 0.56 ± 0.28 Ma younger Pb-Pb age adjustment (Reger et al. 2023). The reported leached bulk-rock compositions of Erg Chech 002 (^238^U/^235^U from 137.81 to 137.83) are higher than the mean proposed for chondrules, chondrites, and achondrites of ∼137.79 (Connelly et al. 2012). Regardless of the origin of U isotopic variation between minerals in achondrites (i.e., ^247^Cm decay or isotopic fractionation), the U isotopic composition of acid washes of mineral separates or whole-rock should be systematically measured to calculate corresponding Pb-Pb isochron ages, because it may lead to an age adjustment up to ∼0.6 Ma in achondrites (Reger et al. 2023; Huyskens et al. 2025).

With the increased number of SLR and LLR chronometry studies of CAIs, individual chondrules and basaltic achondrites, Desch et al. (2023b) used a statistical approach (based on three angrites, two ungrouped NC achondrites NWA 7325 and Asuka 881394, and two ungrouped CC achondrites NWA 6704 and 2976) to constrain the age of the solar system (t_0_) to a value of 4568.4 ± 0.2 Ma. Two key assumptions were made in this study: (i) homogeneous distribution of ^26^Al and other extinct radionuclides, and (2) no inclusion of the U-corrected Pb-Pb ages of CAIs. For all NC achondrites, a systematic lower Pb-Pb age of 0.2 Ma was used based on correlations of estimated pyroxene modal abundances and the measured whole-rock U isotopic compositions following the recommendation of Tissot et al. (2017). Other achondrites such as NWA 7325 (paired with NWA 8486) also indicate a correlation, but this is hampered by large uncertainties on the Pb-Pb ages due to the low U contents of these meteorites, resulting in a less-radiogenic Pb isotopic composition. Consequently, the U isotopic compositions of the samples could not be measured and are assumed to correspond to the mean of achondrites ∼137.79 (Connelly et al. 2012; Goldmann et al. 2015). Using a similar approach based on D’Orbigny Al-Mg and U-corrected Pb-Pb systematics, Piralla et al. (2023) also proposed an older age of ∼ 4568.7 +0.8/−0.6 Ma for solar system formation. Such older t_0_ age than the reported ages for CAIs implies that CAI Pb-Pb ages may have been reset by later thermal events or that ^26^Al was heterogeneously distributed between CAIs and achondrites’ respective formation regions (Schiller et al. 2015a).

### The Quest for a Robust Short-Lived Radionuclide Anchor

Due to remaining ambiguities to reconcile CAI and angrite age records, other possible anchors for ^26^Al-^26^Mg systematics have been investigated among NC achondrites. Three ungrouped achondrites (GRA 06128, NWA 11119, Erg Chech 002) with evolved and alkaline-rich composition (e.g., Srinivasan et al. 2018; Barrat et al. 2021) sample NC asteroids and indicate even earlier crust formation (within 2 to 3 Ma after CAIs) compared to NC basaltic angrites and eucrites (∼3 to 10 Ma after CAIs). They can therefore potentially provide constraints on some of the earliest reservoirs of planetesimal formation and their corresponding initial ^26^Al abundance. Additionally, two ungrouped CC achondrite grouplets (NWA 011/NWA 2976 and NWA 6704) provide a comparison between the NC and CC reservoirs and their ^26^Al abundance. The U-corrected Pb-Pb age of the paired NWA 7325 and NWA 8486 ungrouped NC achondrites is, however, not precise enough and was based on an average U composition for achondrites that may affect its accuracy (4563.9 ± 1.7 Ma; Desch et al. 2023b).

**Fig. 8:**
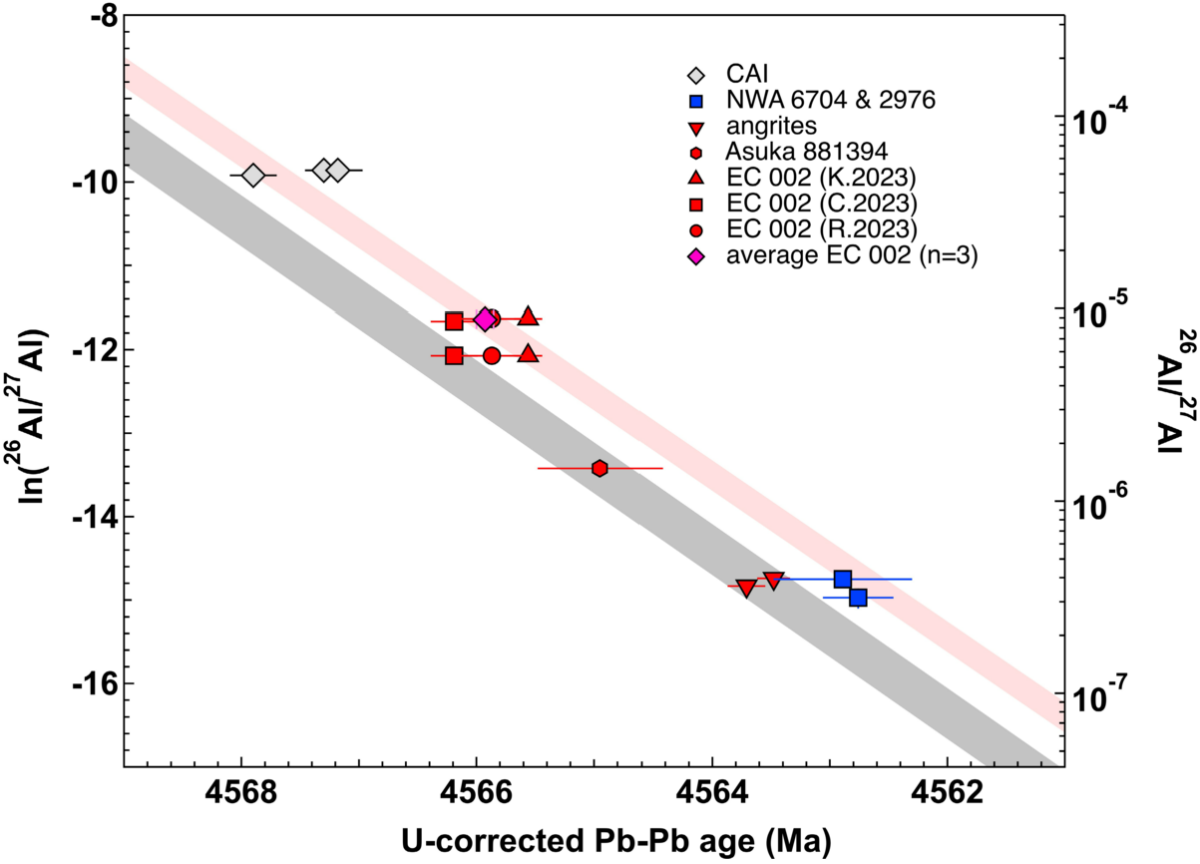
The ^26^Al/^27^Al ratio (in natural log scale) at the time of isotopic closure vs. ^238^U/^235^U-corrected ^207^Pb-^206^Pb ages of individual CAIs (Amelin et al. 2010; Bouvier et al. 2011; Connelly et al. 2012), and NC achondrites (Sahara 99555 and D’Orbigny angrites, Asuka 881397, EC 002, and CC achondrites NWA 6704 and NWA 2976). For EC 002, the U-corrected Pb-Pb ages from three independent studies (Reger et al. 2023; Connelly et al. 2023; Krestianinov et al. 2023) are shown both against the ^26^Al/^27^Al ratio obtained by MC-ICP-MS measurements (∼8.65 × 10^−6^ by Connelly et al. 2023 and ∼8.89 × 10^−6^ by Fang et al. 2022 and Reger et al. 2023) and by in-situ SIMS (∼5.72 × 10^−6^) measurements, respectively. NC- and CC-type achondrites are shown in red and blue, respectively, except for the weighted average Pb-Pb age of EC 002, which is indicated in pink. The shaded areas represent the decay of ^26^Al (slope of −0.967) over time using EC 002 weighted average (pink) or angrite (grey) Pb-Pb ages as anchors, respectively. Modified after Krestianinov et al. (2023)

One critical meteorite found in 2020 is the ungrouped NC achondrite Erg Chech 002 (EC 002). It is particularly suitable as a potential anchor and for interlaboratory studies. Its diverse mineralogy (Na-rich and K-rich feldspars, pyroxenes, olivine, chromite, phosphate) and enriched elemental composition are advantageous to use multiple isotopic systems and small sample masses. Moreover, it is fairly accessible for investigations due to a large total mass initially reported at 31.8 kg. Several independent studies of the EC 002 have been published indicating the oldest crystallization ages for an achondrite using the Al-Mg, Mn-Cr, and U-corrected Pb-Pb chronometers. The age estimates, however, vary outside their reported uncertainties for the different radionuclide systems. Four reported internal ^53^Mn-^53^Cr isochrons for EC 002 fragments provide initial ^53^Mn/^55^Mn corresponding to formation ages that are either contemporaneous with CAIs to 0.7 ± 0.6, 1.3 ± 0.7, or 1.88 ± 0.42 Ma after CAIs, some of which do not overlap within uncertainties (Anand et al. 2022; Zhu et al. 2022; Anand and Mezger 2023; Yang et al. 2025). All reported bulk fragments have also distinct nucleosynthetic $\varepsilon ^{54}$Cr values ranging from −0.35 ± 0.06 to −1.02 ± 0.16 that indicate a heterogeneous composition of the EC 002 meteorite (Anand et al. 2022; Zhu et al. 2022; Krestianinov et al. 2023; Yang et al. 2025). There is no concurrent report of Mn-Cr and Al-Mg systematics on the same fragment of EC 002. Finally, due to the absence of direct measurement of ^53^Mn/^55^Mn in CAIs and discrepancy between the measured U-corrected Pb-Pb ages of CAIs and modelled t_0_ for the formation of the solar system based on achondrites (Fig. 8),an additional ±1.1 Ma uncertainty must be considered for all Mn-Cr modelled ages. The ^26^Al/^27^Al initial abundances obtained by in-situ SIMS and solution analysis MC-ICP-MS correspond to crystallization ages of ∼2.1 and ∼1.8 Ma after CAIs, respectively. The Al-Mg and Mn-Cr isochron age differences could be associated with the different employed analytical methods or from xenotlithic materials as ^54^Cr heterogeneities were found between individual sample fragments (Yang et al. 2025). For instance, SIMS analyses target individual feldspar grains with high Al/Mg ratios using a small spot size, which depends on accurate standardization for Al/Mg ratios. By comparison, solution MC-ICP-MS analysis has low matrix effects due to the separation of Mg prior to isotopic analysis. This method, however, has to pool and dissolve several milligrams of mechanically separated feldspar grains with a minor proportion of mixed mineral grains such as pyroxenes, which dramatically lowers the measured Al/Mg ratios and corresponding ^26^Mg* excesses. Alternatively, the ∼0.3 Ma age gap may be significant and resolvable. It may correspond to two generations of feldspars such as oligoclase (Gattacceca et al. 2021) (possibly anorthoclase; Barrat et al. 2021) and albititic plagioclase (containing lamellae of K-feldspar), because the latter were potentially preferentially analysed by SIMS. The Pb-Pb ages also vary from 4565.56 ± 0.12 Ma (including whole-rock and mineral separate leachates and residues, using ^238^U/^235^U = 137.829 ± 0.005; Krestianinov et al. 2023), to 4565.87 ± 0.30 Ma (pyroxene leachates and residue, using ^238^U/^235^U = 137.766 ± 0.027; Reger et al. 2023), and 4566.19 ± 0.20 Ma (whole-rock leachates and residue, using ^238^U/^235^U = 137.813 ± 0.010; Connelly et al. 2023). The difference in U-corrected Pb-Pb ages of EC 002 may be explained (similarly to what has been described above for angrites) by the variability in U isotopic compositions measured in unleached and leached whole-rock and leached pyroxene samples (Reger et al. 2023). The difference in measured ^238^U/^235^U ratios of EC 002 whole-rocks and pyroxenes causes a ∼0.6 Ma offset on Pb-Pb age calculations. Additionally, variations in the contribution of the three Pb isotopic components (initial, radiogenic, and common Pb contamination) and their corresponding ^207^Pb*/^206^Pb* compositions may explain the 0.63 ± 0.23 Ma gap for two reported Pb-Pb ages (4566.19 ± 0.20 Ma versus 4565.56 ± 0.12 Ma) (Connelly et al. 2023; Krestianinov et al. 2023) with two similar U isotopic whole-rock compositions (contributing to a difference of only ∼0.17 Ma in the Pb-Pb age correction). Out of the three Pb isotope datasets, two are consistent (Reger et al. 2023; Connelly et al. 2023) and one is distinct (Krestianinov et al. 2023) indicating an additional common Pb-like component. The age differences between the two consistent Pb isotope datasets are related to the selection of the U isotopic composition for Pb-Pb age calculation. Alternatively, the EC 002 parent body may be heterogeneous as shown by nucleosynthetic ^54^Cr variations (Yang et al. 2025) obtained from different samples. The ^54^Cr data of the individual samples analysed for their U-corrected Pb-Pb chronology are, however, not available to evaluate, if they derive or do not derive from the same source material. Combining all the evidence, model ages for canonical CAIs using the corresponding solution U-corrected Pb-Pb and Al-Mg analyses from Reger et al. (2023) and Connelly et al. (2023) yield overlapping ages of 4567.7 ± 0.3 Ma, and 4568.0 ± 0.2 Ma, respectively. The Al-Mg and U-corrected ages of EC 002 and ungrouped CC achondrites (NWA 6704 and NWA 2976) are moreover consistent (Fig. 8). The younger U-corrected Pb-Pb CAI ages (Fig. 8) of 4567.30 ± 0.16 Ma (n = 5) obtained on Allende and Efremovka may represent a late resetting event (possibly related to thermal metamorphism on the CV parent body) as suggested by Desch et al. (2023b). Alternatively, both CC and NC achondrites may instead be consistent with a younger t_0_ that is closer to the unpublished U-corrected age of NWA 6991 compact type A CAI mineral separates with a 4.9 × 10^−5^ initial ^26^Al/^27^Al abundance and U-corrected Pb-Pb age of 4567.9 ± 0.30 Ma (Bouvier et al. 2011; Wadhwa et al. 2014).

The U isotopic composition of EC 002 leached whole-rock was measured by three independent laboratories and yielded a mean of 137.82 for the ^238^U/^235^U ratio (Connelly et al. 2023; Reger et al. 2023; Krestianinov et al. 2023). This value is higher than the average composition of ∼137.79 proposed for achondrites (Connelly et al. 2012). This value is also higher than the U isotopic compositions reported for angrites from both petrological groups with 137.80 ± 0.01 and 137.77 ± 0.01 for two quenched angrites and four plutonic angrites, respectively (Tissot et al. 2017). These measurements therefore argue against using a mean value for whole-rock achondrites formed in the NC formation reservoir. Additionally, such resolved variations in ^238^U/^235^U ratios of bulk achondrites add complexities in using bulk U isotopic compositions for pyroxene Pb-Pb dating. We therefore recommend that the U isotopic composition of each bulk or mineral leached sample used for Pb-Pb dating should be measured to avoid possible discrepancy due to U isotopic variation between mineral phases (i.e. as shown in Reger et al. 2023 and discussed in Huyskens et al. 2025).

Alternatively, the systematics of angrites and CAIs can be interpreted in relation to the initial ^26^Al distribution and heterogeneities between planetary formation reservoirs. By comparing the difference of Pb-Pb ages between angrites and CAIs, a lower initial ^26^Al/^27^Al_0_ abundance, as low as (1.3 ± 0.2) × 10^−5^ (∼25% of the accepted canonical value obtained from CAIs), was proposed for the parent reservoir where the angrite parent body formed (Schiller et al. 2015a). For EC 002, Connelly et al. (2023) suggested a value of (2.5 ± 0.6) × 10^−5^ for the initial ^26^Al abundance (∼50% of the canonical value) in its formation reservoir calculated at the time of formation of Efremovka CAIs (Connelly et al. 2012). Using a comparison between EC 002 and angrites, Krestianinov et al. (2023) argued that volcanic angrites indicated a 3 to 4 times lower ^26^Al abundance than EC 002 (Fig. 8). These two independent studies of EC 002 are therefore at odds. Additionally, both EC 002 and angrite parent bodies were formed in the NC region based on Tm and nucleosynthetic ^54^Cr data (Barrat et al. 2021; Zhu et al. 2022). There is therefore a need for additional isotopic and chronological data from pristine achondrites to clarify the initial abundance and distribution of ^26^Al in the inner and outer regions of the protoplanetary disk. Mechanisms must also be proposed to explain how isotopic reservoirs with initial ^26^Al abundances differing by factors of 2–3 could coexist in the NC reservoir (e.g., Iizuka et al. 2025).

The Al-Mg and Hf-W age intervals between CAIs and angrites appear concordant (Kruijer et al. 2014a,b). In addition, the initial ^26^Al/^27^Mg and initial ^182^Hf/^180^Hf ratios correlate for CAIs, CV chondrules, CR chondrules, and angrites, i.e., across different formation regions from both the NC and CC reservoirs (Budde et al. 2018). These important observations argue against a high degree of ^26^Al heterogeneity within the protoplanetary disk in contrast to proposals from several chondrules or achondrite studies (e.g., Larsen et al. 2011; Schiller et al. 2015a; Bollard et al. 2019). The initial ^182^Hf/^180^Hf ratio of EC 002 has not been determined yet for comparison against angrites and CAIs.

The inconsistencies between Al-Mg and U-Pb age differences between CAIs and angrites raise therefore doubts regarding (i) the significance of either U-corrected Pb-Pb ages measured in CAIs or (ii) of both the ^26^Al-^26^Mg systematics and the corresponding Pb-Pb ages of angrites used together as anchors for constraining the initial age (t_0_) of the solar system. Reger et al. (2023) found a good agreement between their results from EC 002 with both the CC achondrite grouplets (NWA 6704 and 2976) and the NWA 6991 B4 CAI, suggesting therefore ^26^Al homogeneity, considering all uncertainties related to the measurements. This interpretation removes the need to call for a large initial ^26^Al heterogeneities at the 30-50% level as previously proposed by angrite and chondrule studies (Schiller et al. 2015a,b; Bollard et al. 2019) or by a EC 002 study (Krestianinov et al. 2023). Although the more limited resolution of the Hf-W system hampers ruling out all ^26^Al heterogeneity, the concordance of these systems indicates that ^26^Al heterogeneity was limited to <20% (Budde et al. 2018), i.e., much less than the ∼80% heterogeneity proposed by e.g., Schiller et al. (2015a).

Radioactive decay systems possess different closure temperatures due to element specific diffusion rates in minerals (Ito and Ganguly 2006; Amelin 2006; Posner et al. 2016). Using a combination of both LLR and SLR systems, planetary events from igneous crystallization to thermal metamorphic processes (cooling or impact-related) can be identified in achondrites. The presence of xenolithic olivine with distinct O isotopic compositions (Rider-Stokes et al. 2023) or higher initial ^26^Al abundances in spinel compared to plagioclase (Deligny et al. 2024) may also explain some of the age inconsistencies observed between mineral and whole-rock Pb-Pb, Al-Mg or Mn-Cr systematics (Fig. 8). Additionally, the assumption that basaltic achondrites cooled rapidly enough to align all LLR and SLR chronometers, and that they evolved without secondary disturbances like thermal metamorphism, aqueous alteration, or impact events, may be inaccurate. A prolonged cooling history could produce some of the age disparities observed with high-precision chronology in achondrites. Thus, it is imperative to further investigate the LLR and SLR systematics in various mineral hosts of EC 002 and other achondrites that crystallized within 5 Ma after CAIs. Such detailed mineralogical records will enable us to detect and document age disturbances and as such to refine the understanding of the formation age of the solar system and the initial ^26^Al abundances within the solar nebula. Such detailed chronological records also provide a way to improve numerical models for thermal evolution, accretion time, and parent body size of meteorites (e.g., Neumann et al. 2023).

## Hf-W Chronometry of Iron Meteorites – Timescales of Accretion and Core Formation on Differentiated Planetesimals

### Iron Meteorites: Remnants of Planetesimal Cores

Vital insights into planetesimal accretion and differentiation come from isotopic studies of iron meteorites. Magmatic iron meteorites consist mainly of metallic iron or Fe-Ni alloys and are interpreted to be fragments of metal cores of protoplanets (Palme and O’Neill 2014; Scott 1972; Scott and Wasson 1975). Consequently, magmatic iron meteorites are the only hand specimens of planetary core material in our collection of extraterrestrial samples and provide an opportunity to directly study the differentiation, cooling, and the crystallization history of the metallic cores of small differentiated solar system bodies. Trace element signatures of iron meteorites indicate that the metal cores of magmatic iron meteorite parent bodies formed by segregation and subsequent cooling and crystallization of metallic melts (Scott and Wasson 1975). The classification of iron meteorites, as well as their formation, crystallization, and cooling history, have been discussed in several comprehensive reviews (Benedix et al. 2014; Chabot and Haack 2006; Goldstein et al. 2009; Scott 2020). Here, we restrict our focus on the accretion and core formation history of iron meteorite parent bodies and their bearing on planetesimal formation and the early evolution of the solar protoplanetary disk.

### Chemical Classification and Solar Nebula Heritage of Iron Meteorites

#### Classification of Iron Meteorites

**Fig. 9:**
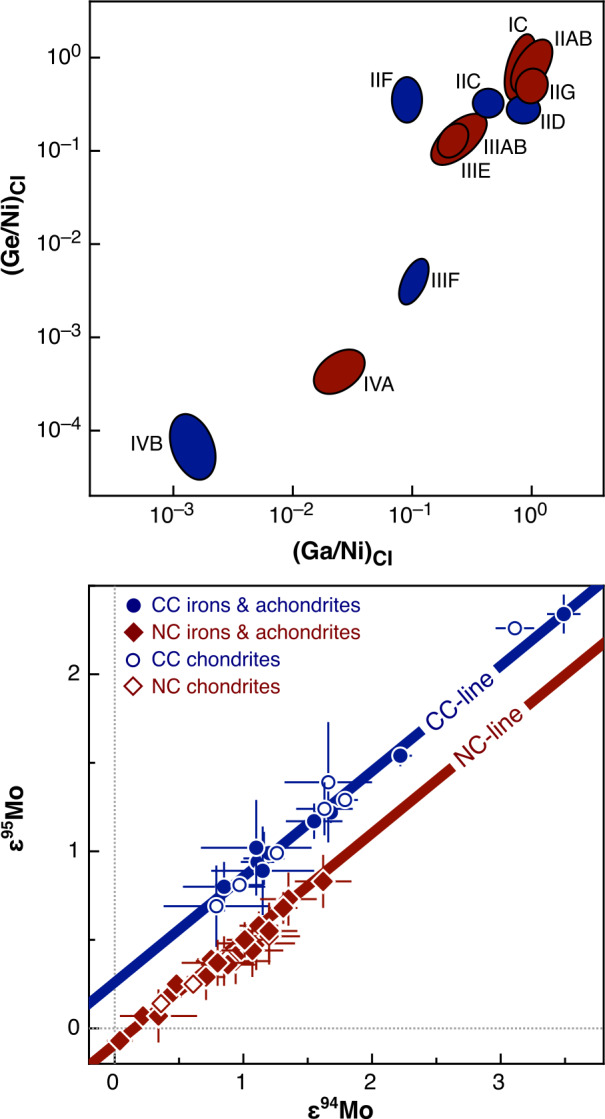
Chemical and isotopic characteristics of iron meteorite groups (a) Volatile element depletions in iron meteorite groups expressed as CI chondrite–normalized Ge/Ni vs. Ga/Ni. (b) Molybdenum isotope dichotomy of iron meteorites and other meteorites expressed as $\varepsilon ^{95} $Mo vs. $\varepsilon ^{94}$Mo where CC (blue) and NC (red) iron meteorite groups define two distinct $s$-process mixing lines with identical slopes but distinct intercept values. Data sources: Goldstein et al. (2009), Scott (1972), Palme and O’Neill (2014), Borg et al. (2022), Budde et al. (2016, 2019), Kruijer and Kleine (2017), Kruijer et al. (2014b), Spitzer et al. (2020). Figure panel b from Kruijer et al. (2020)

Magmatic iron meteorites are classified into several different groups (e.g., IC, IIAB, IIC, IID, IIG, IIIAB, IIIE, IIIF, IVA, IVB) based on their chemical composition, and each group is thought to sample a distinct parent body (Scott and Wasson 1975). Specifically, the groups are primarily distinguished by different contents of moderately volatile elements, such as Ga and Ge (relative to Ni) (Fig. 9a). The order-of-magnitude variations in volatile element depletion may arise from chemical fractionations induced by processes within the solar nebula, suggesting that the conditions of parent body accretion varied in time and/or space, but may alternatively be the result of energetic collisions among iron meteorite parent bodies causing heating and losses of volatile elements. Strong evidence that magmatic iron meteorites formed following crystallization of metallic melt comes from their trace element signatures (e.g., Scott and Wasson 1975). For instance, in a diagram of Ir vs. Ni, the magmatic iron meteorite groups display very well-defined distinct arrays consistent with the trends expected during fractional crystallization of a large volume of metallic melt in planetesimal cores. By contrast, non-magmatic iron meteorites (groups IAB, IIICD, IIE) have trace element abundances that are incompatible with fractional crystallization and, thus, likely do not derive from fully formed planetary cores (e.g., Scott and Wasson 1975; Benedix et al. 2000; Wasson and Kallemeyn 2002; Worsham et al. 2016; Hunt et al. 2018). Instead, the evolution of non-magmatic iron meteorites appears to be more complex, involving metal-silicate separation driven by a combination of internal (endogenic) processes and impact-induced events (Hunt et al. 2018; Worsham et al. 2017). Because of the scientific importance of planetesimal core–derived iron meteorites, this section will mostly focus on magmatic rather than non-magmatic iron meteorite parent bodies. Finally, while most iron meteorites belong to one of the aforementioned groups, there are numerous individual iron meteorites that are chemically distinct and do not fit into any established group - they are referred to as ‘ungrouped’ iron meteorites.

#### Nucleosynthetic Isotope Variations Measured in Iron Meteorites

As discussed in Sect. 1.1, nucleosynthetic isotope signatures allow genetic links between planetary materials to be determined and provide insights into the source regions of meteorites in the solar protoplanetary disk. In particular, the NC-CC dichotomy observed in nucleosynthetic isotope signatures demonstrates that meteorites are derived from two genetically distinct reservoirs. For iron meteorites, the NC-CC dichotomy is most clearly observed in their Mo isotope compositions, where NC iron meteorites (IAB, IIE, IC, IIAB, IIIAB, IIIE, IVA) and CC iron meteorites (IIC, IID, IIF, IIIF, IVB, SBT) plot on two distinct parallel $s$-process mixing lines (Fig. 9b). Other studies have also reported Mo isotopic data for iron meteorites and are generally consistent with the data shown in Fig. 9b. The same Mo isotope dichotomy is also observed for NC chondrites (e.g., ordinary, enstatite) and CC (i.e., carbonaceous) chondrites, as well as other NC and CC achondrites (e.g., Budde et al. 2019; Yokoyama et al. 2019). Additionally, the NC-CC dichotomy for iron meteorites has been observed for multiple elements (e.g., Mo, Ni, Zn, Fe) (Steele et al. 2012; Budde et al. 2016; Kruijer et al. 2017; Poole et al. 2017; Nanne et al. 2019; Yokoyama et al. 2019; Worsham et al. 2019; Spitzer et al. 2020; Hopp et al. 2022a,b; Steller et al. 2022; Martins et al. 2023). A recent study reported additional nucleosynthetic Mo isotope compositions for an extensive suite of ungrouped iron meteorites (Spitzer et al. 2025). These results reveal that Mo isotopic signatures of the ungrouped irons cover the same range of compositions as established iron meteorite groups, indicating that the ungrouped irons can be assigned to either an NC or a CC heritage. Notably, non-magmatic iron meteorites appear to be exclusive to the NC region of the protoplanetary disk. This may be due to the more fragile and porous nature of CC planetesimals, which likely inhibited the formation of impact melt pools (Spitzer et al. 2025).

### Chronology of Metal-Silicate Separation on Magmatic Iron Meteorite Parent Bodies

Precise determination of the timing of core formation for magmatic iron meteorite parent bodies is essential for determining their accretion rates and for identifying the heat sources of differentiation (Kleine et al. 2005; Kleine and Rudge 2011; Neumann et al. 2012; Kruijer et al. 2014b; Lichtenberg et al. 2021), and also has pivotal implications for reconstructing the large-scale evolution of the solar protoplanetary disk (Kruijer et al. 2017; Kleine et al. 2020; Kruijer et al. 2020; Lichtenberg et al. 2021). The core crystallization and cooling history of iron meteorite parent bodies have been studied using long-lived and short-lived chronometers, including the ^187^Re-^187^Os, ^207^Pb-^206^Pb, ^107^Pb-^107^Ag, ^53^Mn-^53^Cr, and ^182^Hf-^182^W chronometers (e.g., Chen and Wasserburg 1990; Shen et al. 1996; Smoliar et al. 1996; Horan et al. 1998; Blichert-Toft et al. 2010; Theis et al. 2013; Matthes et al. 2015; Hunt et al. 2022). A detailed review of the chronology of iron meteorites is beyond the scope of this article but collectively the results imply that their parent body cores crystallized and cooled within a few tens of Ma after CAI formation. To determine the formation and earliest evolution of differentiated planetesimals, it is useful to precisely constrain the time of core formation on iron meteorite parent bodies. The timescales of core formation on differentiated meteorite parent bodies can be constrained using the short-lived and now-extinct ^182^Hf-^182^W system ($t_{1/2} = 8.9$ Ma). This is because both Hf and W are refractory elements but have different geochemical affinities during metal-silicate separation. Because Hf is strongly lithophile and W moderately siderophile, core–mantle differentiation results in high Hf/W in the silicate mantle and Hf/W of essentially zero in the core. Therefore, the Hf-W system is uniquely suited for constraining timescales of core formation on differentiated planetary bodies, provided that core formation occurred within the lifetime of ^182^Hf (see e.g., Harper and Jacobsen 1996; Kleine et al. 2009). In addition, because core formation is relatively closely tied to planetesimal accretion, the Hf-W system also provides indirect insights into the timescales of planetary accretion.

### Application of the Hf-W System to Iron Meteorites

**Fig. 10:**
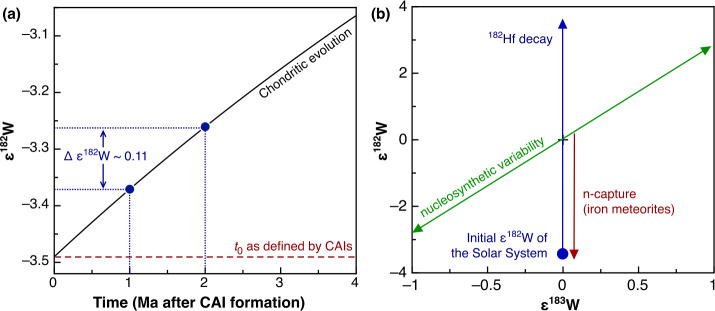
(a) Illustration of two-stage Hf-W model ages for iron meteorites. The measured $\varepsilon ^{182}$W of iron meteorites (blue circles) can be directly compared to the evolution line for chondrites (solid line) to obtain model ages of metal segregation relative to the formation of CAIs (t_0_). (b) Possible processes causing W isotope variations in iron meteorites shown in $\varepsilon ^{182}$W vs. $\varepsilon ^{183}$W space (i.e., ^182^W/^184^W vs. ^183^W/^184^W, internally normalized to ^186^W/^184^W, in parts-per-10^4^ relative to terrestrial standard values). Distinguished are radiogenic contributions from ^182^Hf decay, neutron capture effects during cosmic ray exposure, and nucleosynthetic W isotope variability which results in correlated $\varepsilon ^{182}$W vs. $\varepsilon ^{183}$W variations (Burkhardt et al. 2012; Kruijer et al. 2014a). n-capture refers to neutron capture

The application of the Hf-W system to iron meteorites necessitates the measurement of the ^182^W/^184^W composition of metal samples (commonly expressed in $\varepsilon ^{182}$W as the parts-per-10,000 deviation relative to terrestrial mantle standard values). This is because the metal core, because of its low Hf/W, retains the $\varepsilon ^{182}$W composition at the time of core formation. A two-stage model age of core segregation in the parent bodies of iron meteorites relative to CAI formation can thus be calculated by comparing the measured $\varepsilon ^{182}$W representative of the iron meteorite parent body to the evolution of a reservoir with chondritic ^180^Hf/^184^W (Fig. 10). This is conveniently expressed using the following equation:

4.1 $$ t=\frac{1}{\lambda}ln\biggl(\frac{\varepsilon ^{182}\mathrm{W}_{\mathrm{chondrites}}-\varepsilon ^{182}\mathrm{W}_{\mathrm{SSI}}}{\varepsilon ^{182}\mathrm{W}_{\mathrm{iron}}-\varepsilon ^{182}\mathrm{W}_{\mathrm{SSI}}}\biggr) $$

where $\varepsilon ^{182}$W_iron_ is the measured (radiogenic) value of an iron meteorite, $\varepsilon ^{182}$W_SSI_ is the initial value of CAIs (−3.49 ± 0.07; Kruijer et al. 2014a), $\varepsilon ^{182}$W_chondrites_ is the value of chondritic meteorites, and $\lambda $ is the ^182^Hf decay constant of 0.078 ± 0.002 Ma^−1^ (Vockenhuber et al. 2004). The average measured value of carbonaceous chondrites of −1.91 ± 0.08 is commonly assumed to universally represent the $\varepsilon ^{182}$W of chondrites (Kleine et al. 2004). However, more recently, it has been argued that using a bulk enstatite chondrite-like $\varepsilon ^{182}$W of −2.23 ± 0.13 (corresponding to a ^180^Hf/^184^W of 1.06 ± 0.16) is more realistic for calculating Hf-W model ages of NC iron meteorite groups (Hellmann et al. 2019; Hellmann et al. 2024) instead of the $\varepsilon ^{182}$W value of −1.91 ± 0.08 (and corresponding ^180^Hf/^184^W of ∼1.35) commonly used for CC iron meteorites (Kleine et al. 2004; Kruijer et al. 2014b).

The above approach for calculating two-stage Hf-W model ages assumes that ^182^W variations in iron meteorites are solely radiogenic in origin. However, this assumption is not necessarily accurate because two additional processes, beyond the radiogenic contributions from ^182^Hf decay, can produce ^182^W anomalies in iron meteorites (Fig. 10b). These processes are (i) nucleosynthetic W isotope heterogeneity and (ii) secondary neutron capture effects induced during exposure of the meteoroids to galactic cosmic rays (Leya and Masarik 2013; Leya et al. 2003; Masarik 1997). Nucleosynthetic isotope heterogeneity leads to coupled anomalies in $\varepsilon ^{182}$W and $\varepsilon ^{183}$W, and thus, the magnitude of nucleosynthetic $\varepsilon ^{182}$W variability can be quantified by measuring $\varepsilon ^{183}$W in the same samples (Qin et al. 2008; Burkhardt et al. 2012; Kruijer et al. 2014a). By contrast, quantifying the superimposed effects of secondary neutron capture on W isotopes is more complex as these effects solely appear in (mass bias–corrected) $\varepsilon ^{182}$W. However, as discussed below, accurately quantifying cosmogenic effects is vital for an accurate interpretation of radiogenic $\varepsilon ^{182}$W signatures in iron meteorites.

### Tungsten Isotope Studies of Iron Meteorites and Quantification of Cosmogenic Effects

The earliest comprehensive W isotope study of iron meteorites revealed that they have substantial deficits in ^182^W, indicating that their parent bodies segregated their cores within ca. 5 Ma of each other (Horan et al. 1998). Subsequent, studies conducted at higher precision revealed that iron meteorites exhibit variable ^182^W deficits, which in some cases appeared to be as low as, or even lower than, the initial $\varepsilon ^{182}$W of CAIs (Kleine et al. 2005; Markowski et al. 2006; Scherstén et al. 2006). At face value, these results imply that iron meteorite parent bodies segregated their cores at the beginning of solar system history, i.e., predating the formation of most chondrules and other meteorite parent bodies. However, unaccounted secondary neutron capture effects induced during cosmic ray exposure pose a major issue and cause net downward shift in $\varepsilon ^{182}$W by up to 1 $\varepsilon $, leading to spuriously old Hf-W ages. Initially, several studies employed indirect proxies to quantify these superimposed cosmogenic effects, including published cosmic ray exposure ages and cosmogenic noble gases (Kleine et al. 2005; Markowski et al. 2006; Scherstén et al. 2006; Qin et al. 2008). However, cosmogenic noble gases are mainly produced at higher energies near the surface of a meteoroid, while W isotopes are primarily affected by neutron capture effects at (epi)thermal energies occurring at larger depths (Masarik 1997; Leya and Masarik 2013). Therefore, noble gas proxies only provide an indirect means of quantifying cosmogenic effects on W isotopes. Nevertheless, in some cases, cosmogenic noble gases are helpful in identifying iron meteorite samples with minor to absent neutron capture effects, but such samples are rare (Kruijer et al. 2012). The challenges caused by cosmogenic W isotope effects were overcome with the development of direct neutron capture proxies, including Os, and in particular, Pt isotopes (Kruijer et al. 2013; Wittig et al. 2013). Variations in $\varepsilon ^{196}$Pt (^196^Pt/^195^Pt, internally normalized to ^198^Pt/^195^Pt) are caused solely by cosmic ray–induced neutron capture effects (predominantly on ^195^Pt) and can, therefore, be used to assess the magnitude of neutron capture–induced shifts in $\varepsilon ^{182}$W. The development of direct neutron dosimeters coincided with significant analytical improvements in the precision of W isotope data obtained by MC-ICP-MS (Willbold et al. 2011; Kruijer et al. 2012) and N-TIMS (Touboul et al. 2012) so that the $\varepsilon ^{182}$W of metal samples could now routinely be determined with a precision of ca. 5 ppm (95% conf.).

**Fig. 11:**
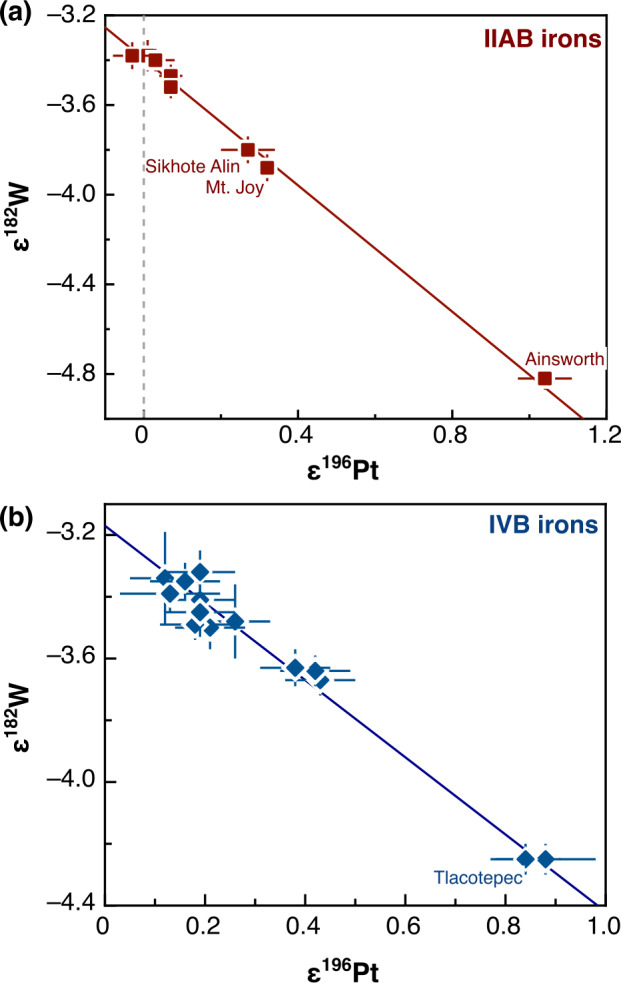
$\varepsilon ^{182}$W vs. $\varepsilon ^{196}$Pt for the (a) IIAB and (b) IVB iron meteorite groups. Solid lines are best-fit regressions through the data. The pre-exposure $\varepsilon ^{182}$W for each group is defined by the intercept of the Pt-W isotope correlation at $\varepsilon ^{196}$Pt = 0. Similar well-defined correlations and precisely determined pre-exposure $\varepsilon ^{182}$W were obtained for other magmatic iron meteorite groups and main-group pallasites (Kruijer et al. 2014b, 2017, 2022). Error bars represent external uncertainties (2 SD for Pt and 95% confidence for W). Figure redrawn after Kruijer et al. (2014b)

Kruijer et al. (2013) reported combined high-precision Pt and W isotope data determined by MC-ICP-MS for the IID and IVB iron meteorite groups that displayed empirical, neutron capture-induced $\varepsilon ^{182}$W vs. $\varepsilon ^{196}$Pt correlations with precise regression-derived pre-exposure $\varepsilon ^{182}$W values for a given group (Fig. 11). Subsequent studies reported combined Pt and W isotope compositions determined by MC-ICP-MS for the major (IIAB, IIIAB, IVA) (Kruijer et al. 2014b) and the minor (IC, IIC, IIF, IIIE, IIIF) iron meteorite groups (Kruijer et al. 2017). Spitzer et al. (2021) identified small nucleosynthetic Pt isotope anomalies in some ungrouped iron meteorites and proposed that the most appropriate pre-exposure $\varepsilon ^{196}$Pt of meteorites is −0.06 ±0.01 instead of zero as was previously assumed. This results in a ca. 0.1 $\varepsilon $ upward correction of the pre-exposure $\varepsilon ^{182}$W values of iron meteorites initially reported in Kruijer et al. (2014b). Most recently, Spitzer et al. (2025) reported W isotope data for an extensive suite of NC and CC ungrouped iron meteorites, significantly increasing the number of iron meteorite parent bodies studied for their W isotope systematics.

While most of the combined Pt-W isotope studies were performed by MC-ICP-MS, others have used W isotopes by N-TIMS in combination with Os isotopes to quantify cosmogenic effects on ^182^W signatures of iron meteorites. This approach was applied to IC iron meteorites (Tornabene et al. 2023), IIC iron meteorites (Tornabene et al. 2020), and the South Byron Trio (SBT) (Hilton et al. 2019). Chiappe et al. (2023) reported combined Pt and W isotopic data for a suite of IIIE irons measured by N-TIMS. Overall, different approaches to correct for cosmogenic effects (Pt vs. Os isotopes) and different mass spectrometry techniques (MC-ICP-MS vs. N-TIMS) have yielded W isotope results and inferred pre-exposure $\varepsilon ^{182}$W for each group that are in excellent agreement.

Pre-exposure $\varepsilon ^{182}$W have also been determined for pallasites (Kruijer et al. 2022), a group of stony-iron meteorites, as well as for the non-magmatic iron meteorite groups, including IAB (Worsham et al. 2017; Hunt et al. 2018) and IIE iron meteorites (Kruijer and Kleine 2019). Kruijer et al. (2022) found that main-group pallasites exhibit pre-exposure $\varepsilon ^{182}$W and Mo isotopic compositions that are indistinguishable from IIIAB iron meteorites. Thus, there is a strong chemical, isotopic, and chronological link between IIIAB irons and main-group pallasites, implying that main-group pallasite metal likely originated in the IIIAB core. Subsequent catastrophic impact disruption of this core would then have caused impact–induced mixing of metal and silicates, resulting in the formation of main-group pallasites (Kruijer et al. 2022; Tarduno et al. 2012). Collectively, the non-magmatic iron meteorites exhibit within-group variations in pre-exposure $\varepsilon ^{182}$W, reflecting a more complex evolution involving endogenic heating and differentiation, followed by impact-induced metal-silicate separation. Consequently, the Hf-W systematics of non-magmatic irons only provide indirect constraints on the accretion and core formation history of their parent bodies and, therefore, will not be further discussed here.

### Pre-Exposure $\varepsilon ^{\textbf{182}}$W and Hf-W Model Ages of Core Formation

**Fig. 12:**
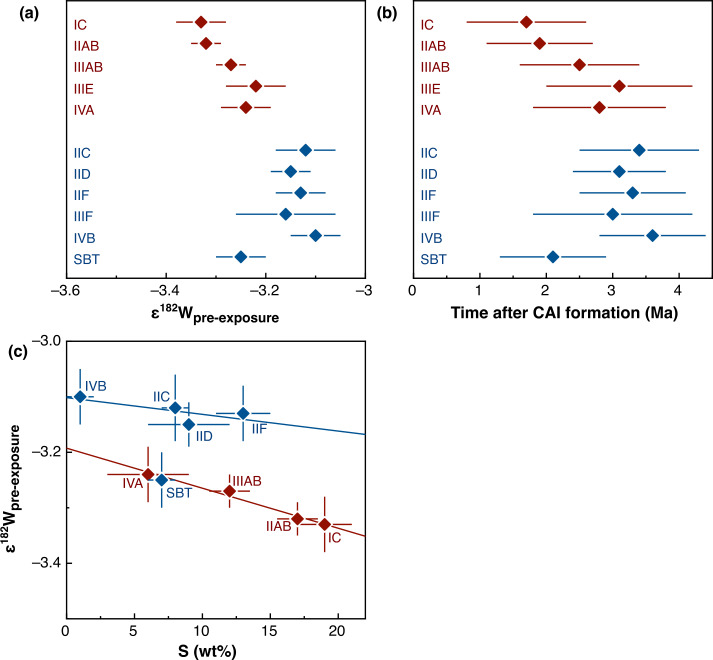
(a) Pre-exposure $\varepsilon ^{182}$W for magmatic iron meteorite groups derived from the NC reservoir (red) and CC reservoir (blue). (b) Two-stage Hf-W model ages of NC and CC iron meteorite groups, where model ages of NC bodies were calculated assuming an enstatite chondrite $\varepsilon ^{182}$W of −2.23 ± 0.13 (Hellmann et al. 2024) but those of CC bodies using a carbonaceous chondrite $\varepsilon ^{182}$W of -1.9 ± 0.1 (Kleine et al. 2004). (c) Pre-exposure $\varepsilon ^{182}$W vs. inferred S concentrations (wt%) for the core of each parent body (see Table S7 in Spitzer et al. 2021 and references therein). Pre-exposure $\varepsilon ^{182}$W are from Kruijer et al. (2014b, 2017), Hilton et al. (2019), Kruijer and Kleine (2019), Chiappe et al. (2023), Tornabene et al. (2023, 2020) and were recalculated assuming a pre-exposure $\varepsilon ^{196}$Pt of −0.06 ± 0.01 (Spitzer et al. 2021)

A summary of the pre-exposure $\varepsilon ^{182}$W of the magmatic iron meteorite groups alongside the corresponding two-stage Hf-W model ages of core formation is given in Fig. 12. Several key observations can be made. First, small but resolvable differences in pre-exposure $\varepsilon ^{182}$W exist between different magmatic iron meteorite groups with a total range of about 0.25 $\varepsilon $ (Kruijer et al. 2014b, 2017; Hilton et al. 2019), where the IC irons (NC) have the lowest $\varepsilon ^{182}$W (−3.34 ± 0.03) and the IVB irons (CC) have the highest $\varepsilon ^{182}$W (−3.10 ± 0.05) (Fig. 12a). After taking into account cosmogenic effects, there are no iron meteorite groups with $\varepsilon ^{182}$W lower than the CAI initial as was the case before cosmogenic effects could be adequately quantified. Second, while both the NC and CC reservoirs exhibit variable pre-exposure $\varepsilon ^{182}$W, the CC iron meteorite groups have, on average, higher and more uniform $\varepsilon ^{182}$W than the NC iron meteorites (Kruijer et al. 2017; Hilton et al. 2019). This translates to a range in two-stage Hf-W model ages of metal segregation ranging of ∼1.5 to 3 Ma after CAI formation for NC iron meteorite groups (assuming an enstatite chondrite like ^180^Hf/^184^W) and a somewhat higher but a more uniform range of ∼3-4 Ma for CC iron meteorite groups (assuming a carbonaceous chondrite-like ^180^Hf/^184^W) (Fig. 12). In addition, with a few exceptions, the $\varepsilon ^{182}$W and corresponding Hf-W model ages of both NC and CC ungrouped iron meteorites generally overlap with those of the iron meteorite groups in each reservoir (Spitzer et al. 2025). Third, pre-exposure $\varepsilon ^{182}$W are inversely correlated with the degree of moderately volatile depletion and the S content inferred for each core (Kruijer et al. 2014b; Hilton et al. 2019; Spitzer et al. 2021). The relation between $\varepsilon ^{182}$W and S contents is most evident for the NC iron meteorite groups, while CC iron meteorite groups define a shallower and somewhat less well-defined correlation (Fig. 12c). Given that the melting temperature of Fe-Ni cores is strongly influenced by their bulk S contents (Wasson and Huber 2006), the $\varepsilon ^{182}$W vs. S correlation suggests that the time of core formation is related to the melting temperature of the parent body (Kruijer et al. 2014b). Specifically, in S-rich bodies (e.g., IIAB, IID), most of the metal melted relatively early at the Fe-FeS eutectic (∼1250 K), resulting in (on average) earlier core formation times. By contrast, in volatile-poor bodies (i.e., IVA, IVB), most of the Fe metal would only have melted after the extraction of silicate melt (at ∼1800 K), resulting in (on average) later times of core formation. This conceptual model can successfully explain the extent of $\varepsilon ^{182}$W variability seen among NC iron meteorite groups and perhaps also among individual CC iron meteorite groups (Fig. 12c). A corollary of this finding is that the Hf-W timescales of core formation on iron meteorite parent bodies, and by inference modelled accretion times, are most reliably assessed on the basis of $\varepsilon ^{182}$W compositions measured in volatile-rich groups (e.g., NC groups IC and IIAB; CC groups IID, IIF) (Kruijer et al. 2017). In addition to the relationship between pre-exposure $\varepsilon ^{182}$W and bulk S content, CC iron meteorite parent bodies have, on average, higher pre-exposure $\varepsilon ^{182}$W than NC iron meteorite bodies, translating into calculated Hf-W ages that are ∼1 Ma younger (Fig. 12a, 12c). This becomes evident when comparing the $\varepsilon ^{182}$W of parent bodies with similar S contents (and volatile contents) (Fig. 12c). Specifically, volatile-rich CC bodies (e.g., IIC, IID, IIF) exhibit pre-exposure $\varepsilon ^{182}$W that are on average ∼0.2 $\varepsilon ^{182}$W higher than volatile-rich NC bodies (e.g., IC, IIAB) (Kruijer et al. 2017). Thus, different melting temperatures of the NC and CC parent bodies cannot (solely) explain the apparent small difference in Hf-W ages between the NC and CC reservoirs. This observation also extends to NC and CC ungrouped iron meteorites (Spitzer et al. 2025). Varying origins for the ∼1 Ma difference in Hf-W ages between NC and CC bodies have been proposed, including (i) earlier accretion times in the inner (NC) compared to the outer (CC) protoplanetary disk (Kruijer et al. 2017; Hilton et al. 2019), (ii) more oxidizing conditions and higher water ice contents in CC compared to NC bodies resulting in delayed times of core formation in CC over NC bodies (Spitzer et al. 2021), and (iii), higher bulk Hf/W in CC compared to NC bodies resulting in distinct $\varepsilon ^{182}$W for identical times of core formation (Kruijer et al. 2014b; Hellmann et al. 2019; Hellmann et al. 2024).

Through a comprehensive Hf-W isochron study of enstatite chondrites, Hellmann et al. (2024) demonstrated that (bulk) enstatite chondrites, and by inference, other NC chondrite precursors, have lower Hf/W than carbonaceous chondrites, and illustrated that enstatite chondrites are a good estimate for the average Hf/W of primitive chondritic material from the inner (NC) disk. Consequently, the lower Hf/W of enstatite chondrites may also be more representative of NC iron meteorite bodies. Using a (time-integrated) $\varepsilon ^{182}$W of −2.23 ± 0.13 determined for enstatite chondrites yields Hf-W model ages that are about 0.4-0.7 Ma younger than those obtained using a carbonaceous chondrite-like Hf/W (Fig. 12b), at least in part, explaining the $\varepsilon ^{182}$W differences between NC and CC iron meteorite groups, and closing the gap in Hf-W model ages for the parent bodies. However, although not fully resolved in all cases, (volatile-rich) CC bodies still exhibit slightly younger Hf-W ages than (volatile-poor) NC bodies, which could either reflect a later accretion time or a delayed onset of melting and core formation in CC bodies compared to NC bodies.

Estimates of the accretion timescales of iron meteorites parent bodies are obtained by combining their Hf-W model ages of core formation with thermal models for bodies heated by ^26^Al decay (t$_{1/2} = 0.7$ Ma) (Hevey and Sanders 2006; Qin et al. 2008; Kruijer et al. 2014b; Hilton et al. 2019; Kaminski et al. 2020). Using a relatively simple thermal model involving conductive heating (Kruijer et al. 2017), this approach yields unequivocally early but slightly distinct accretion ages of <0.5 Ma for NC iron meteorite bodies and 0.5-1 Ma for CC bodies.

A later accretion time for CC compared to NC iron meteorite parent bodies would be consistent with the idea that accretion rates in the outer protoplanetary disk are expected to be nominally slower than in the inner disk. However, Spitzer et al. (2021) noted that CC iron meteorite parent bodies have systematically lower Fe/Ni than their NC counterparts, suggesting that the conditions of core formation were slightly more oxidizing in CC compared to NC iron parent bodies. This is an important observation given that CC bodies likely formed at or beyond the snowline (e.g., Kruijer et al. 2017; Lichtenberg et al. 2021; Izidoro et al. 2022; Morbidelli et al. 2022), allowing them to incorporate water ice. The presence of water ice may have delayed melting on CC parent bodies mainly by reducing the concentration of heat-producing ^26^Al. Additionally, it would have promoted oxidation, resulting in core formation under more oxidizing conditions in CC bodies compared to NC bodies, explaining the difference in observed Fe/Ni. Subsequent work revealed that NC bodies also have significant oxidized Fe in their mantles (Grewal et al. 2024), resulting in overlapping ranges of fO_2_ among NC and CC iron meteorite parent bodies. Nevertheless, using a slightly different approach for estimating oxidation states, Spitzer et al. (2025) found that, although the fO_2_ values of CC and NC iron meteorite parent bodies still partially overlap, the average fO_2_ of CC bodies is slightly but significantly higher than that of NC bodies, consistent with higher water ice fractions in CC over NC bodies.

Using a more advanced thermal model that considers variable water ice fractions, Spitzer et al. (2021) found that a higher water ice fraction indeed results in a delayed onset of melting and metal segregation because it lowers the concentration of heat-producing ^26^Al and enhances heat transfer through water convection. This thermal modelling suggests that, despite slightly different core formation ages, the NC and CC bodies accreted contemporaneously, within <1 Ma after CAI formation (Kruijer et al. 2017; Spitzer et al. 2021). The uncertainties associated with the Hf-W data, and especially with the thermal models used for determining accretion ages, currently do not allow any putative differences in accretion time for these objects to be resolved.

**Fig. 13:**
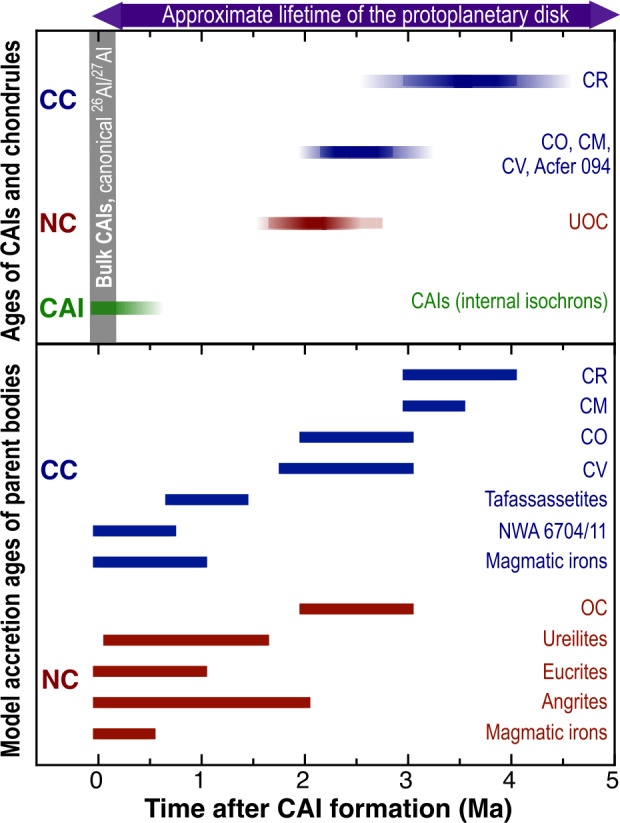
Summary of chronological constraints on early Solar System evolution, distinguishing materials derived from NC and CC reservoirs. Upper panel displays ages of CAIs and chondrules derived from unequilibrated ordinary chondrites (UOC), and various carbonaceous chondrite groups (CV, CO, CM, CR, Acfer 094), determined using Al-Mg chronometry. All relative ages are displayed relative to the U-Pb ages of CAIs (4567.30 ± 0.16 Ma or 4567.94 ± 0.31 Ma; see main text). The estimated lifetime of the solar protoplanetary disk is indicated for context. Lower panel depicts accretion timescales of meteorite parent bodies, inferred from radiogenic ages of meteorites. Accretion ages of iron meteorite and achondrite parent bodies are inferred from model ages for differentiation combined with thermal modelling for internal heating of the parent bodies by ^26^Al decay (see main text). Accretion timescales for chondrite parent bodies are based on Al–Mg, Hf–W and Pb–Pb ages obtained for chondrules (see panel above and corresponding references in main text), and on the chronology of alteration products combined with thermal modelling. Horizontal bars reflect uncertainties in accretion age estimates, not the duration of accretion. Lower panel was modified after Kruijer et al. (2020)

Finally, the accretion time inferred for iron meteorite parent bodies is earlier than the time that most chondrite parent bodies accreted, implying that differentiated planetesimals preceded that of un-melted planetesimals (Fig. 13). This finding is consistent with the notion that ^26^Al decay constituted the primary heat source driving planetesimal differentiation in the early solar system. Specifically, differentiated parent bodies (e.g., like those of iron meteorites) accreted sufficiently early for ^26^Al decay to be an efficient heat source for melting and differentiation, whereas chondrite parent bodies accreted too late for ^26^Al-induced melting to occur (Kleine et al. 2005; Hevey and Sanders 2006; Kleine and Rudge 2011).

The Hf-W chronometry of iron meteorites has far-reaching implications for our understanding of the large-scale evolution of the solar protoplanetary disk. The finding that both NC and CC iron meteorite parent bodies accreted within <1 Ma implies that planetesimal accretion occurred very early (within <1 Ma) at distinct radial locations and in both the inner (NC) and outer (CC) solar protoplanetary disk (Fig. 13). Therefore, the early accretion ages of iron meteorites imply that the spatial separation between the inner (NC) and the outer (CC) solar system was already present within less than 1 Ma after solar system formation (Kruijer et al. 2017) (also see Sect. 1.1 and 5). In addition, core formation in NC and CC bodies likely occurred over slightly distinct timescales (Fig. 12b) because of different oxidation states and water ice fractions in the NC and CC reservoirs (Spitzer et al. 2021). Nevertheless, the moderately volatile element abundances in iron meteorites (e.g., Scott and Wasson 1975) indicate that both the NC and CC reservoirs contained planetesimals with highly variable degrees of volatile element depletion (Kruijer et al. 2014a,b, 2017). Somewhat unexpectedly, the most extreme volatile element depletions appear to be more common in the CC reservoir than in the NC reservoir when ungrouped iron meteorites are included in the analysis (Spitzer et al. 2025). The inference that volatile-poor and volatile-rich NC and CC bodies accreted concurrently rules out the possibility that strongly volatile element depleted iron meteorite parent bodies (e.g., IVA, IVB) accreted earlier than less volatile depleted bodies (e.g., IIAB, IID) (Kruijer et al. 2014a,b). This implies that variable depletions in moderately volatile elements among iron meteorites do not reflect possible increasing condensation of moderately volatile elements in the protoplanetary disk over time. Instead, they must reflect more local processes within the protoplanetary disk, such as localized physical removal of the gas phase during condensation (e.g., Sengupta et al. 2022; Vollstaedt et al. 2020), parent bodies accreting variable contributions of volatile-rich and volatile-poor precursors (e.g., Hellmann et al. 2020; Nie et al. 2021), or secondary, catastrophic impact disruptions of iron meteorite parent bodies causing magma degassing and/or vaporization (e.g., Yang et al. 2007; Horan et al. 2012; Matthes et al. 2018).

## Genetic Relationships and Life Spans of Reservoirs in the Solar Protoplanetary Disk

### Lifetime of Rings in the Protoplanetary Disk

**Fig. 14:**
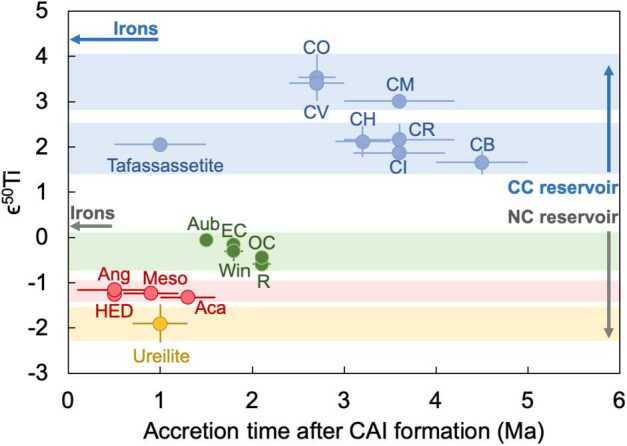
Titanium isotope compositions versus accretion times of various meteorite groups. Accretion ages have relatively large uncertainties and depend on model assumptions. Data from Rüfenacht et al. 2023; accretion ages from Sugiura and Fujiya 2014; Ma et al. 2022; Neumann et al. 2024). The coloured bars show the reservoirs/rings defined by Rüfenacht et al. (2023). Black horizontal arrows indicate the accretion intervals determined for magmatic iron meteorites in the CC and NC regions (see Sect. 4). For abbreviations see Fig. 1

Iron meteorites provide evidence for coeval planetesimal formation and differentiation in the inner (NC) and outer (CC) disk region (Sect. 4, Fig. 13). The combined record of nucleosynthetic isotope compositions indicates further sub-reservoirs within these two regions (Fig. 1; Rüfenacht et al. 2023). This could potentially reflect (i) temporal isotopic changes in the disk over the time span of planetesimal accretion and/or (ii) different formation location in the disk with distinct compositions that remained largely isolated over time. In the scenario of temporal variations, correlations of the accretion ages with the nucleosynthetic isotope compositions are expected (e.g., Sugiura and Fujiya 2014). The nucleosynthetic Ti and Cr isotope data are among those, which exhibit the largest variations relative to their analytical uncertainties, thus providing the highest resolution for silicate materials. Considering the Ti isotope compositions versus accretion time of various meteorite groups (Fig. 14), bodies with nucleosynthetic compositions similar to CR chondrites formed over a period of ∼1 Ma (Tafassasset parent body; Ma et al. 2022) to ∼4 Ma (CR chondrite parent body; Sugiura and Fujiya 2014; Neumann et al. 2024). The CR-associated CB chondrites are of impact origin (Krot et al. 2005; Bollard et al. 2015). If the Ti isotope compositions indeed represent distinct rings in the protoplanetary disk where planetesimals formed, this implies that the compositions of the rings stayed approximately constant from <1 to ∼4 Ma in the CR region with an increasing amount of material locked in planetesimals/asteroids. This further argues for limited exchange between the ring reservoirs to preserve these distinct compositions.

Within these reservoirs, even smaller heterogeneities exist (Fig. 1, Fig. 14). For example, it was argued that Tafassasset and CR chondrites, despite their overall strong isotopic similarities, might have accreted material with slightly different isotopic compositions (Ma et al. 2022). Moreover, CR chondrites fall in the same area as CI chondrites in the ^50^Ti-^54^Cr isotope graph (Fig. 1), but these chondrite groups are clearly distinguishable and well resolved from each other based on ^54^Fe and Ni isotope data (Steele et al. 2012; Schiller et al. 2020; Hopp et al. 2022a,b). Thus, there are nucleosynthetic heterogeneities within the broader reservoirs defined by Ti-Cr isotope data (Fig. 1, Fig. 14). Despite these small-scale heterogeneities within the reservoirs, the nucleosynthetic data are consistent with long-lasting reservoirs in the disk with limited dust exchange between each other. Some minor exchange is indicated, e.g., by NC chondrules found in later formed CC chondrites (Marrocchi et al. 2022; Hertwig et al. 2019; Schrader et al. 2020; Williams et al. 2020). Moreover, fine dust (<10-100 μm) is generally well coupled to the gas and is able to move through the gaps (e.g., Hutchison et al. 2022). This fine dust fraction originated from the outer disk regions and moved inwards with the gas during the later stages of the nebula (Weidenschilling 1997; Desch et al. 2018; Hutchison et al. 2022). This material could be represented in our collections of extraterrestrial materials by CI chondrites (e.g., Schiller et al. 2020) and CI-related samples returned from the rubble-pile asteroids Ryugu and Bennu, because they mostly contain fine-grained material (Yokoyama et al. 2023; Lauretta et al. 2024; Barnes et al. 2025), originally coming from regions beyond where carbonaceous chondrites formed. However, the proportion of small, freely moving dust in the disk, which is enriched in presolar grains, must have remained approximately constant in each reservoir over time to preserve the, at first order, approximately constant isotopic composition of the bodies that formed within each reservoir.

This reasoning for static rings over time can be extended to evidence from Fe, Ni and Mo isotope data (Steele et al. 2011, 2012; Nanne et al. 2019; Worsham et al. 2019; Schiller et al. 2020; Hopp et al. 2022a,b). Compared to lithophile elements like Ti, these elements offer the advantage of exhibiting both lithophile and siderophile tendencies, and thus these data can be obtained for stony, stony-iron and iron meteorite groups. This expands the range of meteorite groups for direct comparison. The resolution offered by these systems (e.g., Fe, Ni) is generally more limited because the isotopic variations are often only marginally larger than the analytical uncertainties. Nevertheless, Ni and Fe isotope data (Steele et al. 2012; Nanne et al. 2019; Schiller et al. 2020; Hopp et al. 2022a,b) reveal distinct similarities between (i) CV-CO-CM chondrites and the magmatic irons IID, IIIF, IVB and between (ii) OCs and the iron meteorite groups IIAB, IIIAB and IVA. The Mo isotope data generally supports these links, but allow for additional heterogeneity, although CM chondrites are difficult to assess because the CM Mo data point seems to be an outlier based on correlation relative to Zr and Ru isotope data (Akram et al. 2015; Fischer-Gödde et al. 2015). In summary, Fe, Ni and Mo isotope data are consistent with the reservoirs proposed based on lithophile elements (Rüfenacht et al. 2023) and also suggest small additional heterogeneities within the reservoirs. The indicated relationships between chondrites and magmatic iron meteorite groups combined with accretion ages (Fig. 14) provide evidence for prolonged accretion in the CV-CO-CM region of <1–3 Ma and in the OC region of <1–2.2 Ma. Considering the overall dichotomy, the isotopic evidence from both lithophile and siderophile elements in chondrites (CR, CV-CO-CM and OC regions) and achondrites suggests extended and coeval time periods during which accretion occurred: <1 to 2.2 Ma in the inner (NC) disk, and <1 to 4 Ma in the outer (CC) disk, possibly reflecting rings in the disk with relatively little dust transfer.

### Size Sorting of Dust as Source of Nucleosynthetic Variations?

Generally, within one region, later formed bodies (chondrites and primitive achondrites) tend to have lower ^50^Ti abundances, although this is at the limit of what can be resolved (Fig. 14). Taken at face value, this hints at the possibility of a change in isotopic compositions over time within a ring. This can be considered as evidence of dust sorting in the rings (Hutchison et al. 2022; Hellmann et al. 2023; Rüfenacht et al. 2023), although the influence of parent body processing (Yokoyama et al. 2011; Goderis et al. 2015), late infall (Van Kooten et al. 2016; Nanne et al. 2019) and selective destruction of presolar grains (Trinquier et al. 2009; Akram et al. 2015) or material with solar system compositions (Ek et al. 2020) in hotter regions closer to the Sun cannot be entirely excluded. Nevertheless, rings formed due to pressure bumps in the protoplanetary disk (e.g., Dullemond et al. 2018), which preferentially trap larger particles, thereby naturally leading to dust sorting (Hutchison et al. 2022). It has been recognized for some time that aerodynamic size sorting of chondrules led to the similar size ranges of chondrules and refractory inclusions observed in each chondrite group (Cuzzi et al. 2001; Jones 2012). Moreover, carbonaceous chondrites are a mixture of matrix, chondrules and CAIs with each component having distinct isotopic compositions (Zanda et al. 2006; Hellmann et al. 2020; Morton et al. 2024). Hellmann et al. (2023) proposed that the variable mixtures of CAIs, chondrules and fine-grained matrix in CV, CO and CM chondrites can be explained by the aerodynamic properties of these components, which affect their preferential incorporation into chondrite parent bodies during planetesimal formation by streaming instability. This results in the formation of matrix-poor bodies first, and later bodies incorporate more fine-grained material from the same region. This scenario can explain, for example, the higher $\varepsilon ^{50}$Ti values and the older accretion age of the CAI and chondrule-rich CO and CV relative to CM chondrites (Fig. 1). This is due to the fact that the $\varepsilon ^{50}$Ti enrichments in carbonaceous chondrites are strongly influenced by the presence of CAIs and chondrules (Leya et al. 2008, 2009; Trinquier et al. 2009; Williams et al. 2020). Hutchison et al. (2022) used a different approach to model nucleosynthetic isotope variations. Presolar grains with their extreme isotope compositions are typically small (< 10 μm) in chondrites, while larger dust particles exhibit more solar-like isotope compositions. Neglecting grain growth, 3-D simulations of disks suggest that trapping larger dust in pressure bumps can cause nucleosynthetic isotope variations within the rings, because they affect the mixing ratios of small presolar and larger solar system-like grains and aggregates (Hutchison et al. 2022). These different dust mixtures can translate into the distinct isotope compositions of planetesimals formed in these regions.

## Summary

Small solar system bodies are key to understanding planet formation because they set the stage for collisional planetary accretion after the gas of the protoplanetary disk has dissipated. Pebble accretion is only possible for larger bodies while the nebular gas is still present. The accretion and differentiation of planetesimals during the initial few Ma of our solar system represent a crucial phase that strongly influenced the final compositions of the terrestrial planets. Here we reviewed the initial stages of planet formation starting from small nebular particles to planetesimal accretion and differentiation using key evidence derived from the study of meteorites.

Valuable insights into the dust of the protoplanetary disk can be obtained from CAI and chondrules using the U-Pb and Al-Mg chronometers. CAIs exhibit a notable range of ^235^U/^238^U ratios. These variations can skew the Pb-Pb ages by up to a few Ma unless precise U isotope measurements are performed on the same samples (Brennecka et al. 2010). Published data on CAIs from CV3 chondrites, along with corresponding U isotope analyses, show a narrow range of U-Pb ages, with an average value of 4567.30 ± 0.16 Ma (Amelin et al. 2010; Connelly et al. 2012). However, a report of an internally U-corrected Pb-Pb isochron on mineral separates indicates a slightly older age of 4567.94 ± 0.31 Ma (Bouvier et al. 2011).

The initial (^26^Al/^27^Al)_0_ ratio of CAIs varies by 20-30% from the canonical value. This implies either an extended period of CAI formation (∼0.3 Ma) or initial ^26^Al heterogeneity among CAIs. Nevertheless, the evidence is consistent with the idea that the ^26^Al abundance in the early solar system was likely homogeneous across both the inner and outer disk regions. It is important to note that the U-Pb and Al-Mg decay systems in chondrules might be easily disturbed by low degree thermal and aqueous alteration because of the ease of self-diffusion in the most relevant host phases (i.e., plagioclase, glass, spinel). Moreover, this diffusion highly depends on the specific chemical compositions of these phases. The ages derived from the U-Pb and Al-Mg systems can also be affected by recycling and reheating in the protoplanetary disk. The initial (^26^Al/^27^Al)_0_ ratio of chondrules, determined from plagioclase isochrons of pristine (types ≤3.05) chondrites seems to be relatively robust and indicates a systematic shift in the chondrule formation location over time, moving from the inner to the outer disk, assuming a homogeneous ^26^Al distribution. The ages suggest chondrule formation occurred at ∼2 Ma after CAIs in the OC region, at 2.2-2.8 Ma in the major carbonaceous chondrite-forming region, and at ≥2.8 Ma in the CR region. Chondrule formation may be linked to the growth of protoplanets at different locations. Earlier formation of chondrules (<1.7 Ma after CAIs) is indicated e.g., by Pb-Pb ages and cannot be fully ruled out, although the U-Pb and Al-Mg chronometers of these chondrules were potentially disturbed due to internal heating of their parent bodies or thermal recycling in the disk.

The detailed chronology of differentiated basaltic achondrites serves as a powerful approach to date planetary processes from igneous crystallization to thermal metamorphism. The age record shows that these processes were well underway within the first few Ma of our solar system, while the solar nebula was still present. Such detailed chronological records help constrain the early planetary thermal evolution, accretion times, and meteorite parent body size.

The age record of basaltic achondrites, however, reveals substantial inconsistencies e.g., between mineral and whole-rock Pb-Pb, Al-Mg or Mn-Cr systematics. This can have several reasons. First, radioactive decay systems possess different closure temperatures due to element-specific diffusion rates in minerals. Hence, slow cooling will inevitably lead to different ages depending on the chronometers used. The assumption that basaltic achondrites cooled quickly enough to align the ages of different chronometers, and that they evolved without secondary disturbances such as thermal metamorphism, aqueous alteration, or impact events, may be inaccurate and thus could account for some of the observed age disparities. Second, the presence of xenolithic olivine (Rider-Stokes et al. 2023) or higher initial ^26^Al abundances in spinel (Deligny et al. 2024) may also contribute to the observed age difference. Third, due to the sensitivity of Pb-Pb ages to the corresponding U isotopic composition, it is recommended to analyse the U isotopic composition of each leached sample to prevent differences caused by U isotope variations between mineral phases. In summary, further chronological investigations on the various host minerals of basaltic achondrites that crystallized within 5 Ma after CAI formation are crucial. This will help identify age disturbances and refine our understanding of the formation age of our solar system and the initial abundance and distribution of ^26^Al in the solar nebula.

Isotopic studies of iron meteorites provide vital insights into planetesimal accretion and differentiation. Based on nucleosynthetic isotope data it has become apparent that iron meteorites derive from two distinct regions within the solar protoplanetary disk, with some parent bodies originating from the inner (NC) disk and others from the outer (CC) disk. The timescales of core formation on iron meteorite parent bodies have been determined using the short-lived ^182^Hf-^182^W decay system. Accurate application of Hf-W chronometry requires highly precise W isotope measurements, complemented by Pt isotope analyses to account for cosmic-ray-induced effects on W isotopes. After correcting for cosmogenic effects, the pre-exposure ^182^W values exhibit slight variability: NC iron meteorite groups exhibit lower ^182^W values and earlier core formation ages (1.5–3 Ma after CAI formation) than CC groups with higher ^182^W values and later core formation ages (3–4 Ma after CAI). For NC iron meteorite groups, an inverse correlation is observed between pre-exposure ^182^W and volatile element depletion, including sulfur content. This observation has been interpreted to reflect that S-rich parent bodies underwent earlier core formation due to lower melting temperatures, whereas core formation in volatile-poor bodies was delayed due to their higher average melting temperatures. Moreover, on average, CC groups exhibit higher ^182^W values and younger Hf-W model ages (>∼1 Ma younger than NC groups), which may reflect earlier accretion in the inner (NC) than outer (CC) disk, more oxidizing conditions and higher water ice content in CC over NC bodies, or differences in bulk Hf/W ratios. Regardless of the exact interpretation, thermal modelling based on ^26^Al decay reveals that both NC and CC parent bodies accreted within <1 Ma after CAI formation. Thus, parent body accretion in both, the NC and CC reservoirs, started early, indicating that a spatial separation between the inner NC and outer CC regions was established within <1 Ma. Moreover, differentiated planetesimals—such as those that produced iron meteorites—accreted earlier than the parent bodies of chondrites. Chondrites formed too late for ^26^Al-induced melting and differentiation to occur. These findings highlight the critical role of ^26^Al as the primary heat source driving early planetary differentiation.

The combination of accretion ages with nucleosynthetic data indicates that, at first order, reservoirs in protoplanetary disks are a long-lived feature with limited exchange between the different reservoirs. Evidence suggests that in the CR chondrite accretion region, new planetary bodies accreted over at least 3 Ma, whereas in most other regions formation occurred over at least 1 Ma with only minor variations in nucleosynthetic isotope compositions over time. Accretion took place during coeval time periods of < 1 to 2.2 Ma in the inner (NC) disk, and < 1 to 4 Ma in the outer (CC) disk. Accepting a disk where large grains (<100 μm) are trapped in pressure bumps forming ring structures and planetesimal formation by streaming instabilities in these rings, implies that size sorting of dust becomes inevitable. Size sorting likely introduced or amplified nucleosynthetic isotope variations now observed between different planetary bodies. These isotope variations may additionally be affected by late infall (Van Kooten et al. 2016; Nanne et al. 2019), selective thermal destruction of presolar grains (Trinquier et al. 2009) or destruction of material with solar system compositions (Ek et al. 2020) in hotter regions closer to the Sun. The latter scenario is particularly attractive for explaining the tight correlation that is observed for many isotope systems (e.g., Cr, Ti; Fig. 1) for inner (NC) disk materials. These correlations are indicative of two-component mixing (Ek et al. 2020; Palme and Mezger 2024). As discussed in Ek et al. (2020), the parental molecular cloud may have contained abundant refractory dust grains with organic-rich icy mantles (Allamandola et al. 1999). Supernova shockwaves in the ISM can implant refractory elements into these mantles (King et al. 2012). Unlike silicates, these volatile mantles are more susceptible to photoevaporation, allowing a broader temperature range for thermal processing in the disk while preserving refractory presolar grains. Removing these icy ISM-grown mantles will affect the isotope mixing ratio of the two-component mixture, while the elemental ratios stay nearly constant. This process naturally accounts for the observed trends. Moreover, removing any material with solar system composition - from icy mantles grown in the ISM to sulfides, metal or silicates - will lead to the observed correlation between nucleosynthetic data of different elements.
